# Diabetes quality management in Dutch care groups and outpatient clinics: a cross-sectional study

**DOI:** 10.1186/1756-0500-7-497

**Published:** 2014-08-07

**Authors:** Marjo JE Campmans-Kuijpers, Caroline A Baan, Lidwien C Lemmens, Guy EHM Rutten

**Affiliations:** 1 Julius Centre for Health Sciences and Primary Care, University Medical Centre Utrecht, Heidelberglaan 100, 3508 GA Utrecht, the Netherlands; 2 Centre for Nutrition, Prevention and Health Services, National Institute of Public Health and the Environment, A. van Leeuwenhoeklaan 9, 3721 MA Bilthoven, the Netherlands

**Keywords:** Diabetes care, Quality management, Quality improvement, Diabetes care group

## Abstract

**Background:**

In recent years, most Dutch general practitioners started working under the umbrella of diabetes care groups, responsible for the organisation and coordination of diabetes care. The quality management of these new organisations receives growing interest, although its association with quality of diabetes care is yet unclear. The best way to measure quality management is unknown and it has not yet been studied at the level of outpatient clinics or care groups. We aimed to assess quality management of type 2 diabetes care in care groups and outpatient clinics.

**Results:**

Quality management was measured with online questionnaires, containing six domains (see below). They were divided into 28 subdomains, with 59 (care groups) and 57 (outpatient clinics) questions respectively. The mean score of the domains reflects the overall score (0-100%) of an organisation. Two quality managers of all Dutch care groups and outpatient clinics were invited to fill out the questionnaire.

Sixty care groups (response rate 61.9%) showed a mean score of 59.6% (CI 57.1-62.1%). The average score in 52 outpatient clinics (response rate 50.0%) was 61.9% (CI 57.5-66.8%).

Mean scores on the six domains for care groups and outpatient clinics respectively were: ‘organisation of care’ 71.9% (CI 68.8-74.9%), 76.8% (CI 72.8-80.7%); ‘multidisciplinary teamwork’ 67.1% (CI 62.4-71.9%), 71.5% (CI 65.3-77.8%); ‘patient centeredness’ 46.7% (CI 42.6-50.7%), 62.5% (CI 57.7-67.2%); ‘performance management’ 63.3% (CI 61.2-65.3%), 50.9% (CI 44.2-57.5%); ‘quality improvement policy’ 52.6% (CI 49.2-56.1%), 50.9% (CI 44.6-57.3%); and ‘management strategies’ 56.0% (CI 51.4-60.7%), 59.0% (CI 52.8-65.2%). On subdomains, care groups scored highest on ‘care program’ (83.3%) and ‘measured outcomes’ (98.3%) and lowest on ‘patient safety’ (15.1%) and ‘patient involvement’ (17.7%). Outpatient clinics scored high on the presence of a ‘diabetic foot team’ (81.6%) and the support in ‘self-management’ (81.0%) and low on ‘patient involvement’ (26.8%) and ‘inspection of medical file’ (28.0%).

**Conclusions:**

This nationwide assessment reveals that the level of quality management in diabetes care varies between several subdomains in both diabetes care groups and outpatient clinics.

## Background

An increasing number of health care providers are involved in diabetes care. Consequently, optimal collaboration among professionals has become essential for delivering high quality of care [1]. This has led to the development of multidisciplinary diabetes teams using disease management programs. Besides monitoring patient related outcomes and process indicators (reflecting actions of health care professionals), quality management (QM) on an organisational level is receiving growing interest in order to maintain or enhance the delivery of good quality diabetes care [2].

In the Netherlands, with a type 2 diabetes prevalence of 5%, 85-90% of all patients with type 2 diabetes are treated by general practitioners in a primary care setting [3,4]. In recent years, most general practitioners started working under the umbrella of diabetes care groups (DCGs). These DCGs are comparable with accountable care organisations in the United States [5,6] and clinical commission groups in the United Kingdom [7]. As the main contractor of a diabetes care program, DCGs are responsible for the organisation and coordination of diabetes care [8,9]. Apart from general practitioners, DCGs contract other health providers like podiatrists and dieticians. Their diabetes care program is based on the Dutch Diabetes Federation Health Care Standard for type 2 diabetes [10]. In 2011, 97 DCGs, with on average 81 general practitioners [11], were treating 170–23,000 diabetes patients per DCG [4]. In 2010, 15% of all patients in primary care were not treated in a DCG [4]. Patients who need more complex diabetes care are treated by endocrinologists in 104 diabetes outpatient clinics (DOCs) [4]. Endocrinologists hold the final responsibility for a diabetes team, consisting of endocrinologists, diabetes nurses, dieticians, and a special team for the treatment of a diabetic foot [12]. Each DOC treats between 250 and 4,500 diabetes patients. Besides the Dutch diabetes standard, they have special guidelines for treatment of a diabetic foot, retinopathy, and nephropathy [12,13].

**Figure 1:**
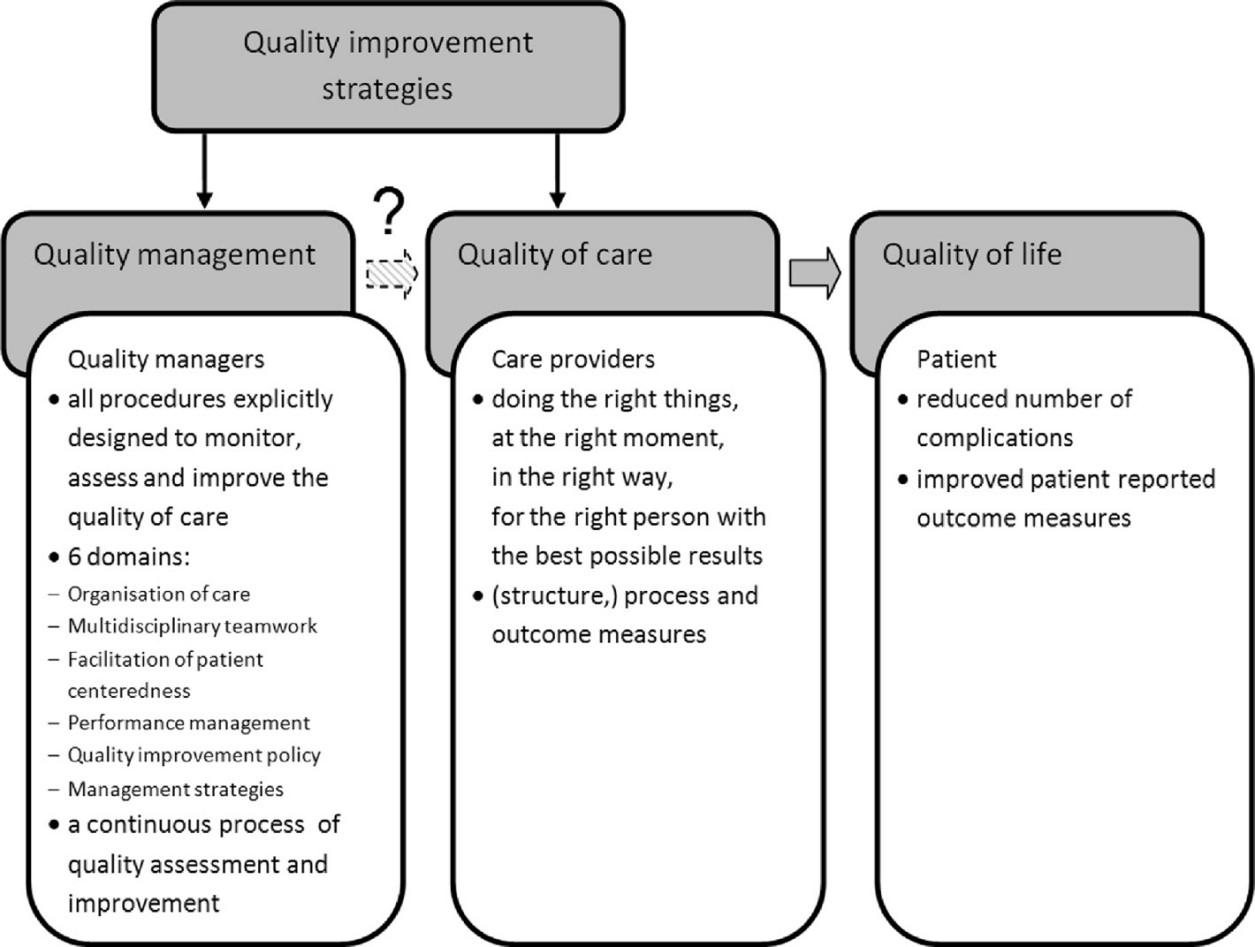
Quality management.

Both DOCs and DCGs need quality management to control the complex diabetes care processes. Quality management comprises procedures to monitor, assess, and improve the quality of care [14]. Validated measures with regard to quality management are lacking. Quality improvement strategies mainly address the individual professional or patient level and generally focus on process and outcomes measures. However, it might be important to focus on the structural or organisational level of diabetes care as well. A meta-analysis of quality improvement strategies showed that interventions upon the *entire* system of chronic disease management, like team changes, case management, continuous quality improvement, or electronic patient registry were in fact associated with the largest effects on HbA1c, irrespective of baseline HbA1c. On the contrary, the effectiveness of interventions targeting individual health care providers and patients seem to vary with baseline HbA1c. Therefore, quality management targeting the entire system should be included in quality improvement strategies for diabetes care [15], which means that these strategies should not only focus on education of the individual health care provider or patient, but address several aspects of the organisation as a whole (see Figure 1). On the other hand a systematic review found that structure indicators, measuring e.g. the adequacy of facilities, equipment, logistics, or registration showed no associations with (surrogate) patient outcomes [16]. Diabetes quality management at an organisational level has only been assessed at the hospital level [14,17], but not yet at the DOC level. DCGs vary widely with regard to the type of legal entity, the ownership and the number of employees [9]. The development of DCGs introduced a new management level on top of the management of general practices. This study aims to measure the level of diabetes quality management in DCGs and DOCs across the Netherlands.

## Methods

### Study design

A cross-sectional measurement of the level of quality management in Dutch DCGs and DOCs was performed. No ethical approval was needed, because this study does not meet the criteria for medical human scientific research according to the Dutch legislation [18].

### Measurements

Based on literature we developed two online questionnaires for quality management for DCGs and DOCs separately. The questionnaires contained six domains: 1.‘organisation of care’; 2. ‘multidisciplinary teamwork’; 3. ‘patient centeredness’; 4. ‘performance management’; 5. ‘quality improvement policy’; and 6. ‘management strategies’. Each domain contained subdomains, in total 28 subdomains were addressed with one to six questions; the total number of questions amounts to 59 for DCGs and 57 for DOCs. In the DCG questionnaire, the diabetic foot team was left out. The DOC questionnaire contained a looping to prevent posing irrelevant questions. Both the score in the domains and subdomains range from 0 to 100%. Details of the development of the questionnaires have been described elsewhere [19] and can be found in Additional files 1 and 2 (in Dutch).

First, all organisations were asked who were the two people mainly responsible for quality management within the organisation. In January 2012, these people of all DCGs (n = 97) and DOCs (n = 104) were invited to fill out the questionnaire. After two and four weeks reminders were sent.

To study whether results are generalizable, non-responders were asked how many patients were enrolled in their diabetes program and how non-responders judged their level of quality management. For this judgment a multiple choice question was used with the following options: 1. insufficiently developed; 2. under development; 3. well developed; and 4. excellently developed, including a cyclic quality management policy. From non-responders who also did not respond to this question we retrieved the number of patients treated from the national website about healthcare organisations in the Netherlands [20].

### Scoring of the questionnaires

#### Scoring on question level

Each question had a maximum score of one point [21]. Since there were different types of questions, different scores were used. Some questions had X subquestions; each subquestion could count for a maximum score of 1/X. Furthermore, the score was higher when the developmental stage on an item was higher. This implies that organisations scored zero points, if they had no policy on an item. If they were developing a policy, the score was 0.33 points; if they had an implemented policy, they scored 0.66 points and if this policy was periodically evaluated, the score was one point. In questions in which we assessed the number of care providers involved in a particular item, each involved care provider scored 1/Y to the maximum score of one point. In the latter type of question the maximum score could be reached when a defined number (Y) of care providers was involved.

#### Scoring of subdomains

Each subdomain consisted of one up to six questions. The maximum score of a subdomain was 100 percent. If a subdomain consisted of for example four questions, a four point’s score was equal to 100 percent.

#### Scoring of domains

To weigh the importance of a subdomain within a domain, two expert panels, of DCGs and DOCs respectively, were asked to weigh the subdomains. These weightings showed significant differences between equal weighting of each domain and the weight given by the expert panels [Additional files 3 and 4] [19]. Therefore, all questions together within a subdomain contributed X percent to the maximum score of a domain, where X was the mean weight given by the corresponding expert panel. The mean score of the six domains reflects the overall score in quality management of an organisation. Descriptive data are presented as means (CI) and medians (IQR) if applicable. The complete scores of the questionnaires for DCGs and DOCs can be found in Additional files 3 and 4 respectively.

### Statistical analysis

To test the representativeness of the participating organisations, their number of patients treated was compared with the number of patients treated by non-responders (independent t-test). Besides, their self-assessed level of quality management was described.

By inviting two responders of each organisation the Cohen’s kappa, which measures the agreement between two responders was calculated [22]. Since both questionnaires contained a wide variety of questions with three to seven answering categories with on top of that multiple answering possibilities, the expected agreement by chance is almost zero.

## Results and discussion

### Results

#### Participants’ characteristics

The responders of 60 diabetes DCGs (response rate 61.9%) were managers (36%), quality employees (18%), managing directors (9%), primary care physicians with specialty in diabetes care (10%) and others (27%). Responders on behalf of 52 DOCs (response rate 50.0%) were endocrinologists with specialty in diabetes care (66%), nurses with specialty in diabetes care (22%), managers (7%) and unit leaders (5%). The number of diabetes patients enrolled in DCGs varied between 170 and 23,000, with a mean number of 6,270 (SD 5442); in DOCs the numbers varied between 250 and 4,500; (mean 1,600, SD 789).

### Diabetes care groups

**Figure 2:**
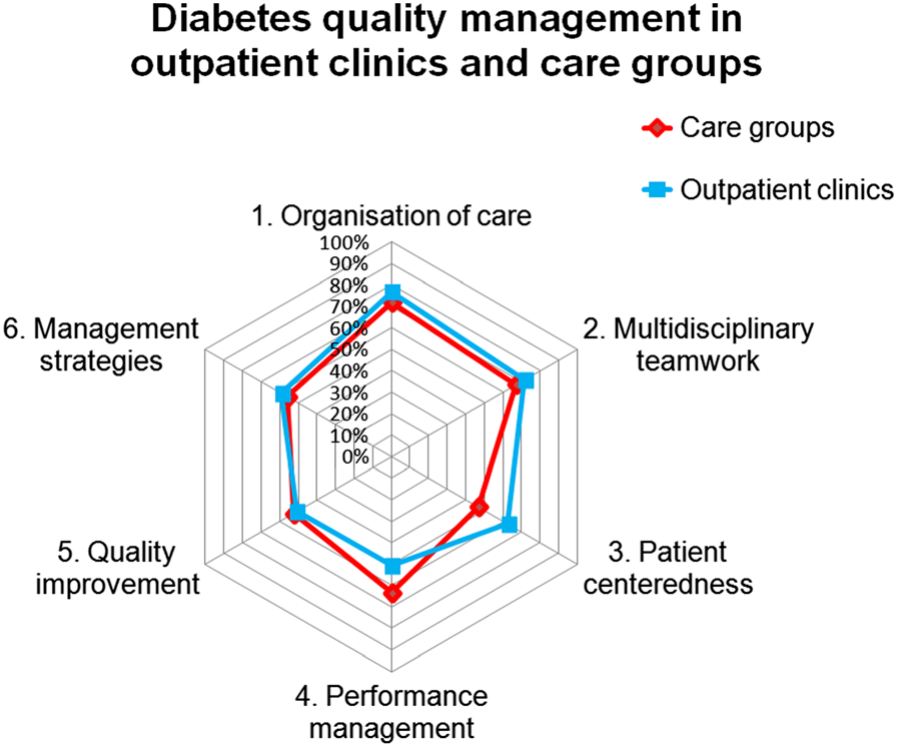
Quality management in diabetes care; score in outpatient clinics and care groups.

DCGs had an overall mean quality management score of 59.6% (CI 57.1-62.1%) (Figure 2; Table 1) with the following mean scores in the domains: ‘organisation of care’ 71.9% (CI 68.8-74.9%), ‘multidisciplinary teamwork’ 67.1% (CI 62.4-71.9%), ‘patient centeredness’ 46.7% (CI 42.6-50.7%), ‘performance management’ 63.3% (CI 61.2-65.3%), ‘quality improvement policy’ 52.6% (CI 49.2-56.1%), and ‘management strategies’ 56.0% (CI 51.4-60.7%).

**Table 1:** The average quality management score on (sub)domains in care groups and outpatient clinics

**Domains and subdomains**	**Care groups (n = 60)**		**Outpatient clinics (n = 52)**	
	**Mean (%)**	**CI (%)**	**Mean (%)**	**CI (%)**
Care program	83.3	80.7-86.0	77.6	73.0-82.2
Continuity and Coordination	65.8	61.0-70.5	76.2	71.6-80.8
Communication and Information	65.5	58.9-72.1	76.5	70.5-82.5
Organisation of care^*^	71.9	68.8-74.9	76.8	72.8-80.7
Work agreement	62.8	59.2-66.4	69.5	61.4-77.5
Tasks and responsibilities	71.1	64.4-77.8	78.6	72.6-84.5
Teamwork/consultation/shared education/guidelines	74.4	68.0-80.7	71.4	63.9-78.8
Transfer and referral	58.5	50.5-66.6	54.2	44.8-63.5
Diabetic foot team	-†	-	81.6	71.3-91.9
Multidisciplinary teamwork*	67.1	62.4-71.9	71.5	65.3-77.8
Self-management	67.9	58.9-76.9	81.0	72.4-89.5
Individual care plan	40.0	33.6-46.4	51.1	41.9-60.4
Policy on patient education	57.3	49.6-65.0	79.2	72.1-86.3
Inspection of medical file	40.4	33.2-47.6	28.0	21.0-35.0
Patient interests	58.2	53.1-63.2	78.1	73.0-83.3
Patient involvement	17.7	12.9-22.4	26.8	19.0-34.7
Patient centeredness^*^	46.7	42.6-50.7	62.5	57.7-67.2
Registering results	59.6	54.5-64.8	57.5	46.7-68.3
Control of results	31.5	25.8-37.2	39.1	29.9-48.2
Processing of results	71.4	66.6-76.1	49.0	41.0-56.9
Analysing results	51.0	46.9-55.1	33.3	24.7-42.0
Measured outcomes	98.3	96.0-100.0	68.8	69.0-77.6
Performance management^*^	63.3	61.2-65.3	50.9	44.2-57.5
Elements of quality improvement	44.5	38.7-50.3	58.5	49.8-67.3
Feedback/benchmark	71.3	66.6-76.1	43.3	34.3-52.4
Visitation	41.4	33.8-49.0	41.7	33.9-49.6
Education	71.6	66.5-76.6	62.8	53.1-72.6
Patient safety	15.1	10.7-19.5	64.8	57.9-71.6
Defining sub-groups	37.8	30.6-44.9	37.9	29.7-46.0
Quality improvement policy^*^	52.6	49.2-56.1	50.9	44.6-57.3
Structural policy	62.9	58.3-67.5	51.5	46.5-56.5
Quality system	36.4	28.3-44.5	63.6	48.8-78.4
Quality documents	55.2	48.5-61.9	67.7	60.2-75.2
Management strategies^*^	56.0	51.4-60.7	59.0	52.8-65.2
*Mean total score:*	59.6	57.1-62.1	61.9	57.5-66.8

*weighted average.

†Care groups do not have a diabetic foot team.

Results of the subdomains demonstrated that DCGs had the highest scores on the ‘care program’ (83.3%; CI 80.7-86.0%) and the ‘measured outcomes’ (98.3%; CI 96.0-100.0%). However, they scored low on ‘patient safety’ (15.1%; CI 10.7-19.5%), and ‘patient involvement’ (17.7%; CI 12.9-22.4%) (Table 1).

### Diabetes outpatient clinics

The overall mean score of the DOCs was 61.9% (CI 57.5-66.8%) (Table 1). Their mean scores in the domains were: ‘organisation of care’ 76.8% (CI 72.8-80.7%); ‘multidisciplinary teamwork’ 71.5% (CI 65.3-77.8%); ‘patient centeredness’ 62.5% (CI 57.7-67.2%); ‘performance management’ 50.9% (CI 44.2-57.5%); ‘quality improvement policy’ 50.9% (CI 44.6-57.3%); and ‘management strategies’ 59.0% (52.8-65.2%). DOCs scored high on the presence of a ‘diabetic foot team’ (81.6%; CI 71.3-91.0%) and the support in ‘self-management’ (81.0%; CI 72.4-89.5%). Their lowest scores were on ‘patient involvement’ (26.8%; CI 19.0-34.7%), and ‘inspection of medical file’ (28.0%; CI 21.0-35.0%).

### Representativeness

From 37 non-responding DCGs 19 answered the non-response question. None reported its level of quality management as ‘insufficient’, six as ‘under development’, seven as ‘good’, and six reported an ‘excellent cyclical quality management’. From 52 non-responding DOCs 30 responded the non-response question. None described its level of quality management as ‘insufficient’, ten as ‘under development’, ten as ‘good’, and ten DOCs reported an ’excellent cyclical quality management’. There was no difference in the number of patients enrolled in the diabetes program between participating and non-participating DCGs (mean 6,270 and 6,690 respectively; p = 0.93). Participating DOCs were larger than non-participating (mean 1,600 and 1,257 respectively; p = 0.02).

### Reliability of the questionnaire

In ten DCGs two responders filled out 59 questions and 196 subquestions. The average observed agreement regarding questions was 69.4% and 71.9% regarding subquestions. This results in Cohen’s kappa values of 0.69 and 0.72 respectively. The questionnaire for DOCs containing 57 questions with 223 subquestions was filled out by two persons in three DOCs. Their average observed agreement level was 64.6% and 63.6% respectively, resulting in Cohen’s kappa values of 0.65 and 0.64 respectively.

### Discussion

This study provides a nation-wide overview of the level of diabetes quality management in diabetes care organisations in the Netherlands. Since this is the first time quality management at this level has been measured, neither a realistic achievable minimum standard nor a benchmark is available. The overall scores on quality management in both types of organisations showed similar results, but the scores on quality management vary between several subdomains in both diabetes care groups and outpatient clinics.

DCGs scored high on ‘organisation of care’ and especially on ‘care program’. This result was not unexpected, because this care program forms the basis of the delivery of their diabetes care. The relatively high scores in ‘performance management’ were not unexpected as well, as process and outcome indicators are being used by insurance companies and patient organisations as a measure for quality of diabetes care delivered by DCGs. Besides, diabetes care providers and DCGs themselves use the results for benchmarking of performance within the DCG. The low score on ‘patient centeredness’ shows that at organisational level the DCGs’ priority is probably not (yet) in this domain. Especially the score on ‘patient involvement’ is low. Indeed patients hardly participate in the decision making regarding content and organisation of disease management within DCGs [23]. There is debate on patient involvement in health care organisations in other countries as well [24]. Apart from a low score on ‘patient safety’, the care group expert panel also weighted this subdomain relatively low (Table 1). Unlike hospital care, primary care has been found to be relatively safe, although incidents do occur in this setting as well [25]. Therefore, patient safety in primary care is likely to receive less priority which might explain the low scores and weighting on ‘patient safety’. One might question whether this is justified or not.

In DOCs, ‘organisation of care’ and ‘multidisciplinary teamwork’ score high; especially the diabetic foot team is well organised. In the Netherlands, people with problems with a diabetes ulcer or a diabetic foot should be referred by general practitioners to DOCs. The last decade, much attention has been paid to the obligatory presence of a diabetic foot team in all Dutch hospitals, with obvious success in terms of avoided amputations [26]. ‘Performance management’ scores low; control and analysing of results need more attention. The lack of appropriate electronic information systems in many Dutch hospitals and the lack of consensus about a minimum set of quality indicators for performance management are likely the main reasons for this result. In 2012, the Netherlands Association of Internal Medicine agreed on a so called e-diabetes core dataset [27] and we may assume that as a result more attention will be paid to ‘performance management’ in the near future. The growing role of health insurance companies requesting performance indicators will also be important in this respect.

To the best of our knowledge, this is the first time the level of quality management has been measured in DCGs and DOCs. If quality management at an organisational level is indeed as important as stated by Tricco [15], our questionnaires might be a useful asset for other diabetes organisations using a disease management program, like accountable care organisations and clinical commission groups. The main issues in quality management for diabetes care are covered and the questionnaires could be used by similar organisations as a basis for their quality improvement programs. However, the proof of concept of quality management at an organisational level to improve diabetes patients’ outcomes in the consulting rooms of the multidisciplinary diabetes team has not yet been established.

A limitation typical of research with self-assessment questionnaires is social desirability. To reduce this social desirability, participating managers were guaranteed that feedback would only be given on their personal email address, thus giving them opportunity to hide this feedback. Furthermore, selection bias may have occurred if organisations with lower levels on quality management were more likely to participate, because they want to learn from this study. On the other hand, organisations with higher levels on quality management might be more willing to participate, as they are eager to learn or like to demonstrate their good level of quality management. Our non-responder question (which was also prone to social desirability) showed equal proportions of organisations which described their level of quality management as ‘under development’ and organisations which described their level of quality management as ‘an excellent cyclical management’ level in both DCGs and DOCs. Furthermore, there is no difference in size between responding and non-responding DCGs, but larger DOCs tend to participate more often. Therefore, we think our data are representative for the quality management level of organisations across the Netherlands.

Another limitation regards the validity and reliability of the questionnaire. Face and content validity were warranted by scrutinising literature for management models and comparing the relevant items from the different models. Also experts from care groups and outpatient clinics were involved in the development of the questionnaires. Next, the corresponding expert-panels weighed the subdomains within a domain. In a pilot study, both draft questionnaires were tested by four and five experts from primary and secondary care respectively. Furthermore, the agreement between two responders of the questionnaires was tested by allowing two respondents of the same organisation to fill out the same questionnaire independently [21]. Although the agreement in the questionnaires could only be tested in ten DCGs and three DOCs, and was often filled out by different type of professionals, the agreement seems to be acceptable. Construct validity was based on literature and a review of seven models for quality management, resulting in the six domains for diabetes quality management [28]. If the questionnaire were to be used for further research, confirmatory factor analyses should be performed. Criterion related validity could not be tested since there were no comparable instruments available.

Participating managers were given feedback on their level of quality management, thus enabling them to improve it [19]. After one year, this level will be measured again. If such an improvement can be achieved (albeit not in a controlled way) and if we could demonstrate a relationship between relevant outcomes of diabetes care and quality management at the organisational level, quality management may become an important instrument in the negotiations between DCGs and DOCs on the one hand and health insurance companies on the other. Until now we can state that quality management varies within DCGs and DOCs and varies between several subdomains.

## Conclusions

This nationwide assessment reveals that the level of quality management in diabetes care varies between several subdomains in both diabetes care groups and outpatient clinics. To study whether quality management and quality of care are associated, quality management and its change needs to be studied further.

## Abbreviations

QM: Quality management; DCGs: Diabetes care groups; DOCs: Diabetes Outpatient clinics.

## Competing interests

The authors declare that they have no competing interests.

## Authors’ contributions

GR and CB were responsible for identifying the QM research question, the design of the study, the acquisition of funding. MC identified the cross-sectional research question, coordinated the study, conducted the analysis and wrote the manuscript. GR, CB, LL helped to draft the manuscript. All authors read and approved the final manuscript.

## Supplementary Material

Additional file 1 Quality management questionnaire for diabetes care groups [In Dutch]. Click here for file

Additional file 2 Quality management questionnaire for diabetes outpatient clinics [In Dutch]. Click here for file

Additional file 3 Scoring of questionnaire for diabetes care groups. Click here for file

Additional file 4 Scoring of questionnaire for diabetes outpatient clinics. Click here for file
