# Supplementary material for: Diabetes quality management in Dutch care groups and outpatient clinics: a cross-sectional study
Source: BMC Res Notes. 2014 Aug 7;7:497. doi: 10.1186/1756-0500-7-497 (PMC4132241; doi:10.1186/1756-0500-7-497)
Supplement: Additional file 1 — Quality management questionnaire for diabetes care groups [In Dutch]. [file 1756-0500-7-497-S1.docx]

| **Vragenlijst kwaliteitsmanagementbeleid**  **diabetes zorggroepen** |
| --- |

| Wij zijn geïnteresseerd in het kwaliteitsmanagement beleid van uw zorggroep. De vragenlijst is bedoeld voor diegene die uitspraken kan doen over het kwaliteitsmanagementbeleid voor diabetes mellitus type 2 van de zorggroep **als geheel**. |
| --- |

| In dit onderzoek definiëren we een ***diabetes* *zorggroep*** als een organisatie met rechtspersoonlijkheid waarin zorgaanbieders zijn verenigd, die verantwoordelijk zijn voor het coördineren en leveren van zorg voor mensen met diabetes type 2 waarbij gebruik wordt gemaakt van een keten-dbc-contract. De zorggroep is de hoofdcontractant van de keten-dbc en levert de gecontracteerde zorg zelf of sluit hiervoor overeenkomsten met individuele zorgaanbieders of – instellingen (onderaannemers). |
| --- |

Is uw organisatie een diabetes zorggroep?

- Onze organisatie is een zorggroep, werkt met een keten-dbc voor diabetes type 2 **of** is hierover in onderhandeling met een zorgverzekeraar
- Nee, de reden hiervan is:
  - Onze organisatie werkt (nog) niet met een keten-dbc voor diabetes type 2 en is ook nog niet in onderhandeling met een zorgverzekeraar
  - De Zorggroep is nog in oprichting. De verwachte oprichtingsdatum is:….
  - Onze organisatie levert voornamelijk AWBZ-zorg
  - Anders, namelijk……

Indien U op bovenstaande vraag nee heeft geantwoord, dan kunt u stoppen met het invullen van de vragenlijst

Hoeveel patiënten met diabetes type 2 worden in uw zorggroep begeleid? ……….

1. Organisatie van zorg

Bij organisatie van zorg komen de volgende onderdelen aan bod:

- Zorgprogramma
- Continuïteit en coördinatie
- Communicatie en informatie

| Een zorgprogramma bevat, naast protocollen over de inhoud van de zorg, ook afspraken over de organisatie en uitvoering van de zorg. | | | |
| --- | --- | --- | --- |
| 1.1 Het zorgprogramma diabetes:  (kies het best passende antwoord) | Ja | In ontwikkeling | Nee |
| Is voor wat betreft het protocol schriftelijk vastgelegd |  |  |  |
| Is inhoudelijk gebaseerd op evidence based standaarden en richtlijnen |  |  |  |
| Is voor wat betreft de afspraken over organisatie en uitvoering schriftelijk vastgelegd |  |  |  |
| Bevat een beschrijving van de taken van *alle* verschillende zorgverleners |  |  |  |
| Is schriftelijk beschikbaar voor de *betrokken* zorgverleners |  |  |  |
| Is digitaal beschikbaar voor de *betrokken* zorgverleners |  |  |  |
| Wordt structureel onderhouden en geactualiseerd |  |  |  |

1.2 Geef aan wie betrokken zijn bij het opstellen, implementeren en/of evalueren en aanpassen van het zorgprogramma diabetes?

| Is betrokken bij het…. van het zorgprogramma  (kruis aan wat van toepassing is, meer antwoorden mogelijk)) | Opstellen | implementeren | evalueren en aanpassen | Niet betrokken |
| --- | --- | --- | --- | --- |
| De directie of het management |  |  |  |  |
| De kader(huis)arts |  |  |  |  |
| Een stuurgroep of commissie |  |  |  |  |
| Een kwaliteitsfunctionaris |  |  |  |  |
| Een extern bedrijf of adviseur |  |  |  |  |
| (Vertegenwoordiger(s) van) zorgverleners |  |  |  |  |
| Vertegenwoordiger(s) van patiënten |  |  |  |  |
| Ander persoon, namelijk ….. |  |  |  |  |

1.3 Hoe vaak wordt het zorgprogramma geëvalueerd?

(kies het best passende antwoord)

- Niet van toepassing. Het zorgprogramma wordt niet geëvalueerd
- Het zorgprogramma wordt niet met een vaste frequentie geëvalueerd
- Jaarlijks
- Tweejaarlijks
- Anders, namelijk……………………………………………………………

1.4 Het zorgprogramma wordt inhoudelijk aangepast als:

(meerdere antwoorden mogelijk)

- Niet van toepassing. Het zorgprogramma wordt niet inhoudelijk aangepast
- NHG standaard is herzien
- NDF zorgstandaard is herzien
- Nieuwe evidence based richtlijnen beschikbaar zijn
- Nieuwe medicatie op de markt komt
- Inhoud van keten-dbc contract is veranderd
- Uitkomsten/ resultaten van zorg daar aanleiding toe geven
- Anders, namelijk……………………………………………………………….

| Continuïteit van zorg houdt in dat er coördinatie van zorg is, uniformiteit in de informatievoorziening naar de patiënt toe en zicht op de in- en uitstroom van patiënten. Daarnaast is een oproepsysteem voor patiënten aanwezig. Zorgverleners moeten kunnen vertrouwen op de uitwisseling van informatie en integratie van zorgactiviteiten. |
| --- |
| De zorggroep dient beleid te ontwikkelen voor de coördinatie van zorg. Zo kan de zorggroep een zorgcoördinator aanstellen ofwel aansturen op een zorgcoördinator per praktijk. Het beleid van de zorggroep kan er ook op gericht zijn de coördinatie van zorg geheel bij de praktijken zelf te laten. |

1.5 Heeft de zorggroep beleid ten aanzien van coördinatie van de zorg?

(kies het best passende antwoord)

- De zorggroep stuurt niet op coördinatie; de praktijken bepalen nu en ook in de toekomst zelf hoe de zorg gecoördineerd wordt
- Coördinatie van zorg is nog geen centraal geleide activiteit van de zorggroep, maar wordt dat in de toekomst wel
- De zorggroep stuurt aan op één zorgcoördinator per praktijk
- De zorggroep heeft zelf een zorgcoördinator, die zorgcoördinatoren binnen praktijken ondersteunt.

1.6 Ontvangen patiënten binnen uw zorggroep naar uw idee uniforme informatie en adviezen van de verschillende zorgverleners over de diabeteszorg?

(kies het best passende antwoord)

- Ja
- Gedeeltelijk
- Nee
- Weet ik niet

1.7 In hoeverre heeft de zorggroep zicht op patiënten die het zorgprogramma in- en/of uitstromen?

(kies het best passende antwoord)

- De zorggroep heeft helemaal geen zicht op de in- en uitstroom van patiënten
- De zorggroep heeft alleen zicht op de instroom van patiënten
- De zorggroep heeft zicht op zowel de in- als uitstroom van patiënten
- De zorggroep heeft alleen zicht op de uitstroom van patiënten

1.8 In hoeverre heeft de zorggroep zicht op de redenen van uitstroom van patiënten?

(meerdere antwoorden mogelijk)

- Niet van toepassing. De zorggroep heeft geen zicht op de uitstroom van patiënten
- De zorggroep heeft geen zicht op de redenen van uitstroom
- De zorggroep weet wie er overleden zijn
- De zorggroep weet wie er verhuisd zijn
- De zorggroep weet wie er naar de tweede lijn zijn gegaan

**1.9 Sommige patiënten komen niet of zeer onregelmatig naar de reguliere controles en ontvangen hierdoor niet de zorg die binnen de zorggroep in het zorgprotocol is afgesproken.**

| (kies het best passende antwoord) | **ja** | **In ontwikkeling** | **Nee** | **Weet ik niet** |
| --- | --- | --- | --- | --- |
| Hebben praktijken hier zicht op? |  |  |  |  |
| Heeft de zorggroep hier zicht op? |  |  |  |  |
| Wordt hierop door de zorggroep actie ondernomen? |  |  |  |  |
| Wordt hierop door de praktijken actie ondernomen? |  |  |  |  |

| Een oproepsysteem is een procedure of applicatie waarvan gebruik gemaakt wordt om patiënten op te roepen voor bijvoorbeeld de jaarcontrole. Dit kan al dan niet centraal binnen de zorggroep geregeld zijn. |
| --- |

1.10 In hoeverre wordt er binnen de zorggroep met een oproepsysteem voor patiënten gewerkt?

(kies het best passende antwoord)

- De zorggroep heeft geen centraal oproepsysteem en laat het oproepen van patiënten geheel aan de praktijken over
- De zorggroep stimuleert het gebruik van een oproepsysteem in de praktijken
- De zorggroep stelt een oproepsysteem voor patiënten verplicht aan alle praktijken
- De zorggroep heeft een centraal oproepsysteem waarmee huisartsen de mogelijkheid hebben hun patiënten centraal op te laten roepen voor de jaarcontrole

| De communicatie binnen een zorggroep kan bevorderd worden door gebruik te maken van een informatiesysteem (bijvoorbeeld HIS of KIS). Naast actuele en juiste gegevens van de patiënt met betrekking tot diagnose, gewenste uitkomsten, afspraken over behandelingen en bereikte doelen, kunnen hierin het individueel behandelplan en zelfmanagementdoelen worden bijgehouden. |
| --- |

1.11 Binnen de zorggroep wordt op dit moment:

(meerdere antwoorden mogelijk)

- In verschillende praktijken met verschillende HISSEN gewerkt
- Met een HIS en KIS naast elkaar gewerkt
- Allemaal in eenzelfde HIS gewerkt
- Allemaal in eenzelfde KIS gewerkt
- Gestreefd om allemaal met dezelfde HIS te gaan werken
- Gestreefd om allemaal met eenzelfde KIS te gaan werken
- anders, namelijk

1.12 Geef aan wat bij u van toepassing is wat betreft het informatie- en communicatiesysteem.

| (kies het best passende antwoord) | **Ja** | **In**  **Ontwikkeling** | **Nee** |
| --- | --- | --- | --- |
| Binnen de zorggroep is één methode van registratie (standaardisatie van bijvoorbeeld zorguitkomsten) |  |  |  |
| In het HIS/KIS zijn mogelijkheden om het individuele behandelplan bij te houden |  |  |  |
| In het HIS/KIS zijn mogelijkheden om het individuele zorgplan bij te houden |  |  |  |
| In het HIS/KIS zijn mogelijkheden om zelfmanagementdoelen bij te houden |  |  |  |
| Worden zorgverleners door het informatiesysteem (HIS/KIS) eraan herinnerd de protocollen te volgen |  |  |  |
| Binnen de zorggroep is eenheid in verwijsformulieren |  |  |  |
| Binnen de zorggroep is eenheid in laboratoriumformulieren |  |  |  |

1.13 Als de zorggroep gebruik maakt van een KIS, welke zorgverleners hebben dan toegang tot het (elektronische) patiëntendossier?

| (kies het best passende antwoord) | **Ja** | **Nee** | **N.v.t. (Niet werkzaam voor de zorggroep)** |
| --- | --- | --- | --- |
| Huisarts |  |  |  |
| POH |  |  |  |
| Diabetesverpleegkundige |  |  |  |
| Internist |  |  |  |
| Oogarts |  |  |  |
| Optometrist |  |  |  |
| Apotheker |  |  |  |
| Diëtist |  |  |  |
| Podotherapeut |  |  |  |
| Fysiotherapeut |  |  |  |
| Anders, nl…………………… |  |  |  |
| Anders, nl…………………… |  |  |  |

# 2. Multidisciplinaire samenwerking

| Multidisciplinaire samenwerking betekent dat verschillende disciplines met hun eigen expertise met elkaar samenwerken, waardoor kennis en zorg rondom een patiënt gebundeld wordt. Meestal worden multidisciplinaire richtlijnen of zorgprotocollen gebruikt. Samenwerkingsafspraken, verwijscriteria, verantwoordelijkheden en bevoegdheden dienen goed vastgelegd te zijn. |
| --- |

- 1. Met welke zorgverleners heeft uw zorggroep schriftelijke werkafspraken gemaakt over het zorgprogramma diabetes ( NB. Hier hoeft u geen contract mee te hebben)

| (aankruisen wat van toepassing is) | **Ja** | **In ontwikkeling** | **Nee** |
| --- | --- | --- | --- |
| Huisarts + POH |  |  |  |
| Diabetesverpleegkundige |  |  |  |
| Internist |  |  |  |
| Oogarts |  |  |  |
| Optometrist |  |  |  |
| Apotheker |  |  |  |
| Diëtist |  |  |  |
| Podotherapeut |  |  |  |
| Psycholoog |  |  |  |
| Fysiotherapeut |  |  |  |
| Anders, namelijk |  |  |  |

2.2 Wat doet uw zorggroep om multidisciplinaire samenwerking te faciliteren?

| (kies het best passende antwoord) | **Ja** | **In**  **ontwikkeling** | **Nee** |
| --- | --- | --- | --- |
| Er is een expliciete beschrijving van de taken en verantwoordelijkheden van betrokken zorgverleners |  |  |  |
| Er zijn afspraken gemaakt over taaksubstitutie (bijvoorbeeld POH-ers die taken van de huisarts overnemen) |  |  |  |
| De zorggroep stimuleert het multidisciplinair overleg over diabetespatiënten (structureel overleg tussen tenminste 2 samenwerkende zorgverleners vanuit verschillende expertise). |  |  |  |
| De zorggroep organiseert periodiek gezamenlijke scholing voor betrokken zorgverleners |  |  |  |

2.3 Welke samenwerking vindt er in uw zorggroep plaats? Is deze vastgelegd in een protocol? Wordt deze samenwerking geëvalueerd en worden de uitkomsten gebruikt om de kwaliteit van de samenwerking te verbeteren?

| (meerdere antwoorden mogelijk) | **Zijn er niet/ vindt niet plaats** | **Is nog niet in protocol vast- gelegd** | **Zijn in protocol vast-**  **gelegd** | **Worden periodiek geëva-**  **lueerd** | **Worden gebruikt bij verbeter-acties** |
| --- | --- | --- | --- | --- | --- |
| De zorggroep heeft afspraken over samenwerking tussen de zorgverleners |  |  |  |  |  |
| De zorggroep heeft afspraken over overdracht van patiënten tussen de zorgverleners binnen de zorggroep |  |  |  |  |  |
| De zorggroep heeft afspraken over overdracht van patiënten naar de zorgverleners buiten de zorggroep |  |  |  |  |  |
| De zorggroep heeft multidisciplinaire afspraken over verwijs- en terugverwijscriteria (bv. naar diëtist en internist) |  |  |  |  |  |
| De zorggroep heeft structureel overleg met zorgverleners over de resultaten van zorg (bijv. spiegelinformatiebijeenkomsten) |  |  |  |  |  |
| De zorggroep organiseert regelmatig bijeenkomsten voor het bespreken van richtlijnen/ standaarden |  |  |  |  |  |
| De zorggroep organiseert regelmatig bijeenkomsten voor het bespreken van taken en verantwoordelijkheden |  |  |  |  |  |
| Anders, namelijk ……………… |  |  |  |  |  |

2.4 De consultatiefunctie van de internist:

(meerdere antwoorden mogelijk)

- Is vastgelegd in het protocol van het zorgprogramma
- Er zijn afspraken gemaakt over de criteria van consultatie
- Er zijn afspraken gemaakt over frequentie van consultatie
- Er is vastgelegd dat consultatie plaats vindt via telefonisch overleg
- Er is vastgelegd dat consultatie plaats vindt via e-mail
- Afspraken worden structureel geëvalueerd en zo nodig bijgesteld

2.5 De consultatie functie van de internist:

(kies het best passende antwoord)

- Wordt niet of te weinig gebruikt, omdat ……….
- Wordt volgens de afgesproken frequenties gebruikt
- Wordt te veel gebruikt, omdat …………..
- Er zijn geen afspraken over frequenties
- Mate van gebruik is onbekend

# 3. Patiëntgerichtheid

Bij patiëntgerichtheid komen de volgende onderdelen aan bod:

- Zelfmanagement
- Individueel zorgplan
- Patiënteducatie
- Inzage gegevens
- Patiëntenbelangen
- Patiëntbetrokkenheid

| Zelfmanagement: is erop gericht de patiënt in staat te stellen eigenmachtig te beslissen en te handelen. Hiervoor moet de patiënt eerst inzicht krijgen in het ziektebeeld door adequate patiënteducatie, inzage in de eigen medische gegevens en wordt er met de patiënt samen een individueel zorgplan opgesteld rekening houdend met diens wensen, mogelijkheden en omstandigheden. Dit individuele zorgplan wordt vervolgens samen met de patiënt regelmatig geëvalueerd en bijgesteld. |
| --- |

Zelfmanagement

3.1 Hoe wordt zelfmanagement bij diabetespatiënten door de zorggroep ondersteund:

(kies het best passende antwoord)

- Vindt niet plaats
- Ondersteuning is momenteel in ontwikkeling
- Verspreiden van informatie (folders, boekjes)
- Cursussen voor zorgverleners
- Cursussen voor patiënten
- Anders, namelijk

Individueel zorgplan

3.2 Het opstellen van individuele zorgplannen

(kies het best passende antwoord)

- Wordt niet gestimuleerd door de zorggroep
- Is in ontwikkeling binnen de zorggroep
- Wordt actief door de zorggroep gestimuleerd
- Wordt actief binnen de zorggroep gestimuleerd en periodiek geëvalueerd aan de hand van vooraf bepaalde doelen

Patiënteducatie

3.3 Het beleid van de zorggroep ten aanzien van patiënteducatie is:

(kies het best passende antwoord)

- Niet ontwikkeld binnen de zorggroep
- In ontwikkeling binnen de zorggroep
- Dat praktijken zelf de patiënteducatie regelen
- Om zoveel mogelijk door middel van afstemming tot een uniforme informatievoorziening te komen
- Om zoveel mogelijk door middel van afstemming tot een uniforme informatievoorziening te komen en zorgverleners hier ook in te trainen

Inzage gegevens

3.4 Hoe heeft de patiënt zelf inzage in zijn/haar medische gegevens?

(kies het best passende antwoord)

- De patiënt heeft alleen inzage in medische gegevens als hij/zij hier uitdrukkelijk om vraagt.
- Toegang tot medische gegevens is in ontwikkeling
- De patiënt kan de gegevens zien in de diabetespas
- De patiënt kan via een patiëntenportaal zijn gegevens inzien
- Anders, namelijk .....

3.5 Kan de patiënt zelf gegevens toevoegen aan zijn elektronische dossier?

(kies het best passende antwoord)

- Nee, er is geen elektronisch dossier
- Er is wel een elektronisch dossier, maar patiënt kan geen gegevens toevoegen
- De mogelijkheid voor de patient om zelf gegevens toe te voegen is in ontwikkeling
- Ja, via een patiëntenportaal

Patiëntenbelangen

| **3.6 Patiënten belangen**  (kies het best passende antwoord) | Ja | In Ontwikkeling | Nee |
| --- | --- | --- | --- |
| Is er in een protocol vastgelegd dat de patiënt geïnformeerd wordt over richtlijnen/standaarden (bv met de diabeteszorgwijzer)? |  |  |  |
| Is er via de website van zorggroep informatie voor patienten beschikbaar? |  |  |  |
| Is er bij de zorggroep een centrale plek (front-Office) waar de patiënt met vragen terecht kan? (een balie, een centraal telefoonnummer of een website) |  |  |  |
| Worden spreekuurtijden van de verschillende hulpverleners op elkaar afgestemd? |  |  |  |
| Is er een vast aangewezen persoon tot wie de patient zich met al zijn vragen kan richten? |  |  |  |
| Wordt de privacy van de patiënt gegarandeerd bij een multidisciplinair zorgdossier? |  |  |  |
| Wordt de privacy van de patiënt gegarandeerd bij het verzamelen van gegevens voor bv de benchmark? |  |  |  |

Patiëntbetrokkenheid

3.7 Hoe worden patiënten betrokken bij uw zorggroep? Door middel van ….

(meerdere antwoorden zijn mogelijk)

- Patiënten zijn niet betrokken bij de zorggroep
- Patiënten zijn nog niet structureel betrokken bij de zorggroep, maar we zijn dit wel aan het voorbereiden
- Cliëntenraad op zorggroep niveau
- Klachtencommissie op zorggroep niveau
- Structurele samenwerking met de regionale patiënten/ consumentenfederatie (NPCF of Zorgbelang)
- Structurele samenwerking met de patiëntenvereniging (DVN)
- Anders, namelijk.……

# 4. Resultaten

Bij resultaten komen de volgende onderdelen aan bod:

- Registreren, verzamelen en verwerken van gegevens
- Indicatoren

| Gegevens worden geregistreerd op structuur-, proces- of uitkomstniveau. Uit de verzamelde data kunnen prestatie- of kwaliteitsindicatoren worden afgeleid om de kwaliteit van zorg te meten en verbeteren. Het is belangrijk hierbij ook patiënt-tevredenheidsindicatoren te gebruiken. |
| --- |

4.1 Hoe worden de geregistreerde gegevens van onderstaande zorgverleners verzameld voor de zorggroep?

| (Kies het best passende antwoord) | Via een elektronisch informatiesysteem | Via een papieren dossier | Worden niet verzameld |
| --- | --- | --- | --- |
| Huisarts + POH |  |  |  |
| Diabetesverpleegkundige |  |  |  |
| Internist |  |  |  |
| Oogarts |  |  |  |
| Optometrist |  |  |  |
| Diëtist |  |  |  |
| Podotherapeut |  |  |  |
| Fysiotherapeut |  |  |  |
| Anders, nl…. |  |  |  |

| Gegevens worden niet altijd juist ingevoerd of verwerkt. Een dataset kan door foutieve invoer onjuiste extreme waarden bevatten. Controle van gegevens is daarom van belang. |
| --- |

4.2 Hoe heeft de zorggroep georganiseerd dat de door de zorgverleners geregistreerde en aangeleverde gegevens op juistheid worden gecontroleerd?

(meerdere antwoorden mogelijk)

- De zorggroep heeft hier niets voor georganiseerd
- Dit gebeurt door de zorgverleners zelf
- De zorggroep besteedt dit uit aan een onafhankelijke organisatie
- In het informatiesysteem zijn waarschuwingen ingebouwd om onjuiste data te voorkomen
- Anders, namelijk......

4.3 Wie bewerkt de aangeleverde resultaatgegevens van de zorgverleners tot feedbackgegevens/interne indicatoren?

(kies het best passende antwoord)

- De betreffende zorgverleners doen dit zelf
- De zorggroep doet dit
- De zorggroep besteedt dit uit aan een onafhankelijke organisatie
- Anders, namelijk......

4.4 Wie bewerkt de aangeleverde data van de zorgverleners tot externe verantwoordingsindicatoren aan bijvoorbeeld Zichtbare Zorg (ZIZO) of verzekeraars?

(kies het best passende antwoord)

- De betreffende zorgverleners doen dit zelf
- De zorggroep doet dit
- De zorggroep besteedt dit uit aan een onafhankelijke organisatie
- Anders, namelijk......

| Resultaatgegevens kunnen op verschillende manieren worden teruggekoppeld. Indien de zorggroep of zorgverlener alleen een gemiddelde van een indicator gepresenteerd krijgt, is dat niet altijd voldoende aanleiding om hier verbeteracties op in te zetten. |
| --- |

4.5 Op welk niveau worden de gegevens geanalyseerd?

(Meerdere antwoorden mogelijk)

- Gegevens worden niet geanalyseerd
- Op patiëntniveau
- Op praktijkniveau
- Op huisartsengroep (Hagro) niveau
- Op zorggroepniveau

4.6 Op welke wijze worden de gegevens geanalyseerd?

(Meerdere antwoorden mogelijk)

- Gegevens worden niet geanalyseerd
- Alleen gemiddelden worden bepaald
- Zowel gemiddelden als spreiding worden bepaald
- Ook individuele extreme waarden worden bepaald
- Op basis van medicatiegebruik worden subgroepen bepaald en op dat niveau wordt geanalyseerd
- Op basis van demografische gegevens worden subgroepen bepaald en op dat niveau wordt geanalyseerd
- Anders, namelijk ….

4.7 Welke dataset wordt in de zorggroep geregistreerd?

(meerdere antwoorden mogelijk)

- Er wordt geen specifieke dataset geregistreerd
- De minimale dataset (MDS) van de Nza, Zichtbare Zorg
- De e-Diabetes kernset van de NDF
- Anders, namelijk …

4.8 Welke indicatoren worden berekend?

(Meerdere antwoorden mogelijk)

- ZIZO indicatoren
- Indicatoren voor verzekeraars
- NHG indicatoren
- Anders, nl. ……….

# 5. Kwaliteitsverbetering

Bij kwaliteitsverbetering komen de volgende onderwerpen aan bod:

- Elementen kwaliteitsverbetering
- Spiegelinformatie en benchmark
- visitatie
- Scholing
- Patiëntveiligheid
- Subgroepen

5.1 Welke resultaten gebruikt uw organisatie voor kwaliteitsverbetering?

|  | **Niet**  **Gemeten** | **Wel gemeten.**  **Gebruikt voor kwaliteitsverbetering?** | |
| --- | --- | --- | --- |
| (kies het best passende antwoord) |  | **ja** | **Nee** |
| **Prestatie-indicatoren** |  |  |  |
| **Ervaringen van de patiënt:** bijvoorbeeld door afname van de CQ-index |  |  |  |
| **Meningen van de verwijzers of andere partners in de zorgketen:** bijvoorbeeld tevredenheidonderzoeken of evaluatierapporten |  |  |  |
| **Gezamenlijke klachtenregistratie:** de zorggroep heeft een overzicht van alle klachten die binnen komen bij de zorggroep |  |  |  |
| **Wachttijden bij zorgverleners:** worden wachtlijsten/wachttijden voor de toegang tot de zorgverleners binnen de organisatie gemeten? |  |  |  |
| **Anders, namelijk ....** |  |  |  |

Spiegelinformatie en benchmark

| Spiegelinformatie is het presenteren en vergelijken van resultaten of indicatoren binnen de organisatie om zodoende de zorg te verbeteren |
| --- |

5.2 Met welke zorgverleners wordt de spiegelinformatie besproken?

(meerdere antwoorden mogelijk*)*

- Niet van toepassing. Er is geen spiegelinformatie aangeboden
- Huisartsen
- Praktijkondersteuners
- Diabetesverpleegkundige
- Diëtisten
- Podotherapeuten
- Oogartsen
- Optometrist
- Internisten
- Psycholoog
- Anders, namelijk………………………………………………………………………………

| Benchmarking is het vergelijken van resultaten en indicatoren met andere organisaties om verbeteringen door te voeren. |
| --- |

5.3 Wordt benchmarking bij uw zorggroep gebruikt om verbeteringen door te voeren:

(kies het best passende antwoord)

- Nee, benchmarking wordt niet gebruikt
- Benchmarking wordt incidenteel gebruikt
- Het beleid om benchmarking structureel in te zetten voor kwaliteitsverbetering is nog in ontwikkeling
- Benchmarking wordt structureel gebruikt om de kwaliteit te verbeteren

Visitatie

| Visitatie is een vorm van intercollegiale toetsing gericht op het functioneren van een individuele collega of een aantal samenwerkende zorgverleners. |
| --- |

5.4 Op basis waarvan wordt een zorgverlener binnen de zorggroep gevisiteerd?

(meerdere antwoorden mogelijk)

- Zorgverleners worden niet door de zorggroep gevisiteerd
- Nieuw toegetreden zorgverleners worden gevisiteerd
- Zorgverleners die slecht scoren op de resultaten van zorg worden gevisiteerd
- Alle zorgverleners worden periodiek door de zorggroep gevisiteerd

5.5 Welke zorgverleners binnen de zorggroep worden periodiek gevisiteerd?

(meerdere antwoorden mogelijk)

- Er vindt geen of slechts incidenteel visitatie plaats
- Huisartsen(praktijken)
- Diëtisten
- Oogartsen
- Internisten
- Podotherapeuten
- Anders, namelijk………………

Scholing

5.6 Hoe is het scholingsbeleid vormgegeven?

(kies het best passende antwoord)

- Niet van toepassing: scholing wordt niet gezien als een verantwoordelijkheid van de zorggroep maar van de individuele zorgverlener
- Het scholingsbeleid binnen de zorggroep is in ontwikkeling
- De zorggroep heeft scholingsbeleid voor een deel van de zorgverleners binnen de zorggroep
- De zorggroep heeft een scholingsbeleid voor alle zorgverleners van de zorggroep
- De zorggroep heeft een scholingsbeleid voor alle zorgverleners van de zorggroep, dat regelmatig wordt geëvalueerd en bijgesteld

5.7 Voor welke zorgverleners heeft de zorggroep in het afgelopen jaar bij- of nascholing georganiseerd?

(meerdere antwoorden mogelijk)

- Niet van toepassing, er is geen bij- of nascholing georganiseerd
- Huisartsen
- POH-ers
- Doktersassistentes
- Internisten
- Diabetesverpleegkundigen
- Diëtisten
- Oogartsen
- Optometristen
- Podotherapeuten
- Anders, namelijk……………..

Patiëntveiligheid

| De hoofdbehandelaar heeft de eindverantwoordelijkheid voor de totale behandeling van de patiënt |
| --- |

5.8 Is in een protocol vastgelegd op welke wijze incidenten moeten worden gemeld?

(kies het best passende antwoord)

- Nee, dit is de verantwoordelijkheid van de individuele zorgverlener
- Nee, dit is in ontwikkeling
- Ja, incidenten worden aan de hoofdbehandelaar gemeld
- Ja, incidenten worden aan de hoofdbehandelaar en aan de zorggroep gemeld

| Zorggerelateerde schade is schade die een patient oploopt als gevolg van een bijwerking van een behandeling of verkeerde handeling in de zorg |
| --- |

5.9 Wordt er binnen de zorggroep een systeem gebruikt dat systematisch scant en waarschuwt wanneer een patiënt mogelijke zorggerelateerde schade kan gaan ondervinden?

(kies het best passende antwoord)

- Nee, dit wordt niet gebruikt
- Dit wordt incidenteel door een aantal zorgverleners binnen de zorggroep gebruikt
- Nee, dit is in ontwikkeling voor alle zorgverleners binnen de zorggroep
- Ja, dit is binnen de hele zorggroep operationeel

Subgroepen

5.10 Worden er binnen uw zorggroep subgroepen onderscheiden om gericht beleid in te kunnen zetten (bijvoorbeeld voor mensen met nierproblemen)?

(Kies het best passende antwoord)

- Nee, dit onderscheid wordt in de zorggroep niet gemaakt
- Hier wordt incidenteel naar gekeken
- In de zorggroep worden structureel subgroepen van patiënten onderscheiden
- Hier wordt structureel naar gekeken en waar mogelijk ook beleid op ingezet

5.11 Wordt er binnen uw zorggroep speciaal beleid ingezet om moeilijk bereikbare en/of complexe patiëntgroepen de juiste zorg te geven?

(Meerdere antwoorden mogelijk)

- Nee, dit vindt niet plaats in de zorggroep
- Ja, voor mensen met een lage sociaal economische status
- Ja, voor mensen van allochtone afkomst
- Ja, voor zorgmijders
- Ja, voor minder mobiele mensen
- Ja, voor mensen die veel verschillende medicijnen gebruiken (polyfarmacie)
- Ja, voor mensen met multimorbiditeit
- Anders, namelijk …

# 6. Kwaliteitsbeleid

Bij kwaliteitsbeleid komen de volgende onderwerpen aan bod:

- Structureel kwaliteitsbeleid
- Kwaliteitssysteem
- Kwaliteitsdocumenten

Structureel kwaliteitsbeleid

6.1 Wie is de voortrekker van het kwaliteitsbeleid in uw organisatie?

(kies het best passende antwoord)

- Niemand
- De kaderarts
- Een stuurgroep of commissie
- De kwaliteitsfunctionaris
- Een extern bedrijf of adviseur
- Vertegenwoordigers van zorgverleners
- De directie of het management
- Anders, namelijk …..

6.2 Op welke manier is het kwaliteitsbeleid structureel ingebed in uw organisatie?

(meerdere antwoorden mogelijk)

- Het is niet structureel ingebed
- Er is een speciaal intern budget gereserveerd voor kwaliteitsbeleid
- Er zijn één of meer kwaliteitsfunctionarissen aangesteld
- Er is een expertteam/ kwaliteitsteam met (kader)huisartsen, diabetesconsulenten en/ of internisten
- Er is een extern bedrijf of adviseur betrokken
- Anders, namelijk………………………………..

| Cyclisch kwaliteitsbeleid wordt vaak gebruikt als middel om voortdurend kwaliteit te verbeteren. Een bekend voorbeeld hiervan is de kwaliteitscirkel van Deming, waarbij de stappen plan-do-check-act (PDCA) worden doorlopen. |
| --- |

6.3 Wordt binnen de zorggroep een vorm van cyclisch kwaliteitsbeleid gebruikt als instrument om de kwaliteit van diabeteszorg te verbeteren?

(kies het best passende antwoord)

- Nee
- Ja, maar incidenteel
- Ja, nu nog incidenteel, maar structureel gebruik is in ontwikkeling
- Ja, we maken daar structureel gebruik van

Kwaliteitssysteem

6.4 Werkt uw zorggroep met een gecertificeerd kwaliteitssysteem?

(kies het best passende antwoord)

- Ja, namelijk:
- ISO
- INK
- HKZ
- Anders, namelijk
- Nee, alleen op huisartspraktijkniveau wordt een kwaliteitssysteem gebruikt
- Nee, de zorgverleners moeten wel voldoen aan specifieke kwalificering voor hun zorgprofessie
- Nee

Kwaliteitsdocumenten

6.5a Kunt u aangeven welke van de volgende documenten uw zorggroep heeft?

| (kies het best passende antwoord) | **Ja** | **In**  **ontwikkeling** | **Nee** |
| --- | --- | --- | --- |
| **Missiedocument:** visie en prioriteiten van de zorggroep |  |  |  |
| **Kwaliteitsactieplan voor de zorggroep:** maatregelen voor de implementatie en de planning van acties om kwaliteitsdoelen te bereiken |  |  |  |
| **Jaarlijks kwaliteitsrapport:** rapportage over alle uitgevoerde activiteiten om kwaliteit te borgen en de resultaten daarvan |  |  |  |
| **Kwaliteitshandboek:** beschrijving van alle procedures die de zorggroep gebruikt voor kwaliteitsborging en de verantwoordelijke personen daarvoor |  |  |  |
| Is het **kwaliteitshandboek** beschikbaar voor alle medewerkers binnen de zorggroep? |  |  |  |

6.5b Valt Uw zorggroep onder de Wet Toelating Zorginstellingen (WTZi)?

(kies het best passende antwoord)

- Ja
- Nee
- Weet ik niet

# Toekomst

Wat zijn de twee belangrijkste prioriteiten in kwaliteitsmanagementbeleid voor het volgende jaar?

(2 antwoorden aankruisen)

- (Verder) formuleren van procedures
- (Verder) formuleren van protocollen
- Kwaliteits(verbeter)projecten aan de hand van spiegelinformatie
- Kwaliteits(verbeter)projecten aan de hand van benchmarking
- Consequent volgen van de kwaliteitscirkel (plan-do-check-act)
- Patiëntervaringen-onderzoeken
- Visitering van een aantal beroepsgroepen binnen de zorggroep
- Accreditatie van een aantal beroepsgroepen binnen de zorggroep
- Certificering van een aantal beroepsgroepen binnen de zorggroep
- Accreditatie of certificering van de zorggroep als geheel
- Verbeteren van ICT mogelijkheden
- Introduceren/verbeteren van mogelijkheden tot zelfmanagement van patiënten
- Competenties en scholing van de zorgverleners op peil brengen/houden
- Verbeteren van de verwijzing/samenwerking met zorgverleners buiten de organisatie
- Anders, namelijk …..

Heeft u nog vragen of opmerkingen over de vragenlijst of anderszins?

- ………

Wie heeft de vragenlijst ingevuld?

- Directeur
- Manager
- Kwaliteitsfunctionaris
- Kaderarts
- Anders, namelijk ....

### Bedankt voor het invullen van de vragenlijst

De vragenlijst kan worden de retourneert in de bijgesloten antwoordenvelop naar:

Marjo Campmans-Kuijpers, MSc

Onderzoeker in opleiding, UMC Utrecht

Locatie Julius Centrum, Stratenum

Huispostnummer: STR: 6.131-Kamernummer: STR 5.122

Postbus 85500, 3508 GA Utrecht

Telefoon: 06-23295934

E-mail: [m.j.e.campmans-kuijpers@umcutrecht.nl](mailto:m.j.e.campmans-kuijpers@umcutrecht.nl)
