# Supplementary material for: Diabetes quality management in Dutch care groups and outpatient clinics: a cross-sectional study
Source: BMC Res Notes. 2014 Aug 7;7:497. doi: 10.1186/1756-0500-7-497 (PMC4132241; doi:10.1186/1756-0500-7-497)
Supplement: Additional file 2 — Quality management questionnaire for diabetes outpatient clinics [In Dutch]. [file 1756-0500-7-497-S2.doc]

| Wij zijn geïnteresseerd in het kwaliteitsmanagement beleid van diabetes mellitus type 2 in uw polikliniek. Bij diabetespoli wordt de poliklinische afdeling van interne geneeskunde of endocrinologie bedoeld. De vragenlijst is bedoeld voor diegene die uitspraken kan doen over het kwaliteitsmanagementbeleid ten aanzien van diabetes type 2 in deze polikliniek. |
| --- |

| **Vragenlijst kwaliteitsmanagementbeleid diabetes mellitus type 2**  **poliklinieken** |
| --- |

Welkom bij de digitale vragenlijst over het kwaliteitsmanagementbeleid diabetes bij poliklinieken

Deze vragenlijst bestaat uit 50 vragen, verdeeld over zes aandachtsgebieden van managementkwaliteitsbeleid voor de behandeling van mensen met diabetes type 2.

Het invullen kost u ongeveer 30 minuten.

Desgewenst kunt u het invullen van de vragen onderbreken om op een later moment terug te keren en de vragenlijst af te ronden. U begint dan automatisch bij de

vraag waar u gebleven was.

Nadat u de vragenlijst volledig heeft ingevuld ontvangt u per e-mail uw resultaten in een spinnenwebgrafiek. U krijgt dan ook toegang tot een online toolbox,waarmee u aan de slag kunt om uw diabeteskwaliteitsbeleid waar nodig te verbeteren.

Meer informatie vindt u op: www.diabeteskwaliteitsbeleid.nl.

Voor vragen neemt u contact op met Marjo Campmans, onderzoeker.

Mail: onderzoek@diabeteskwaliteitsbeleid.nl

Tel: 06-23295934

Deze enquête is onderdeel van het project ‘Aan de slag met kwaliteitsbeleid’ van het Nationaal Actieprogramma Diabetes (NAD).

**1. Organisatie van zorg**

Bij organisatie van zorg komen de volgende onderdelen aan bod:

- Zorgprogramma
- Continuïteit en coördinatie
- Communicatie en informatie

| Een zorgprogramma bevat, naast protocollen over de inhoud van de zorg, ook afspraken over de organisatie en uitvoering van de zorg. |
| --- |

| - 1. Het zorgprogramma diabetes:   (kies het best passende antwoord) | **Ja** | **In**  **ontwikkeling** | **Nee** |
| --- | --- | --- | --- |
| Is voor wat betreft het protocol schriftelijk/digitaal vastgelegd |  |  |  |
| Is inhoudelijk gebaseerd op evidence based standaarden en richtlijnen |  |  |  |
| Is voor wat betreft de afspraken over organisatie en uitvoering schriftelijk vastgelegd |  |  |  |
| Bevat een beschrijving van de taken van *alle* verschillende zorgverleners |  |  |  |
| Is beschikbaar voor de betrokken zorgverleners |  |  |  |
| Wordt structureel onderhouden en geactualiseerd |  |  |  |
| Bevat een beschrijving van alle stappen die de patiënt doorloopt (zorgpad) |  |  |  |

- 1. Geef aan wie zijn betrokken bij het opstellen, implementeren en/of evalueren en aanpassen van het zorgprogramma diabetes?

| (kruis aan wat van toepassing is, meerdere antwoorden mogelijk) | opstellen | Implemen-teren | evalueren en aanpassen | niet betrokken |
| --- | --- | --- | --- | --- |
| De medische staf |  |  |  |  |
| De endocrinoloog of internist met de meeste affiniteit voor diabetes |  |  |  |  |
| Diabetesverpleegkundigen |  |  |  |  |
| Een diabetescommissie |  |  |  |  |
| Een kwaliteitsfunctionaris |  |  |  |  |
| Vertegenwoordiger(s) van patiënten |  |  |  |  |
| Anders, namelijk ….. |  |  |  |  |

1.3 Hoe vaak wordt het zorgprogramma geëvalueerd?

(kies het best passende antwoord)

- Niet van toepassing. Het zorgprogramma wordt niet geëvalueerd
- Het zorgprogramma wordt niet met een vaste frequentie geëvalueerd
- Jaarlijks
- Tweejaarlijks
- Anders, namelijk……………………………………………………………

1.4 Het zorgprogramma wordt inhoudelijk aangepast als:

(meerdere antwoorden mogelijk)

- Niet van toepassing. Het zorgprogramma wordt niet inhoudelijk aangepast
- NIV standaard is herzien
- NDF zorgstandaard is herzien
- Nieuwe evidence based richtlijnen beschikbaar zijn
- Nieuwe medicatie op de markt komt
- Inhoud van keten-dbc contract is veranderd
- Uitkomsten/ resultaten van zorg daar aanleiding toe geven
- Anders, namelijk……………………………………………………………….

| Continuïteit van zorg houdt in dat er coördinatie van zorg, uniformiteit in de informatievoorziening naar de patiënt toe en zicht op de in- en uitstroom van patiënten is. Daarnaast is er een oproepsysteem voor patiënten aanwezig. |
| --- |

1.5 Hoe stuurt de diabetespoli op coördinatie van de zorg?

(kies het best passende antwoord)

- Coördinatie van zorg is geen prioriteit binnen de diabetespoli
- Coördinatie van zorg is in ontwikkeling binnen de diabetespoli
- De diabetespoli heeft één zorgcoördinator per patiënt, namelijk een diabetesverpleegkundige (in overleg met de internist)
- De diabetespoli heeft één zorgcoördinator per patiënt, namelijk de internist
- Anders, namelijk…..
  1. In hoeverre zijn de uitspraken wat betreft continuïteit van zorg op uw diabetespoli van toepassing?

| (kies het best passende antwoord) | helemaal niet  **1** | **2** | **3** | **4** | Helemaal wel  **5** |
| --- | --- | --- | --- | --- | --- |
| De patiënten op de diabetespoli ontvangen eenduidige adviezen van de verschillende zorgverleners m.b.t. de diabeteszorg? |  |  |  |  |  |
| De verwijzing vanuit de 1e lijn naar de diabetespoli verloopt conform regionale transmurale afspraken. |  |  |  |  |  |
| De verwijzing van de diabetespoli naar de 1e lijn verloopt conform regionale transmurale afspraken. |  |  |  |  |  |
| De diabetespoli stimuleert het structureel inplannen van zorgafspraken voor patiënten |  |  |  |  |  |
| De diabetespoli stimuleert het gebruik van een oproepsysteem voor het oproepen van patiënten |  |  |  |  |  |
| De diabetespoli heeft zicht op patiënten die wegblijven bij de behandelaar (verloop van de patiënten) |  |  |  |  |  |
| De diabetespoli stimuleert het gebruik van een herinneringssysteem bij no show contacten |  |  |  |  |  |
| De diabetespoli heeft zicht op patiënten die uitstromen bij de behandelaar (door overlijden, verhuizen, overgang naar eerst lijn) |  |  |  |  |  |

| De communicatie kan bevorderd worden door gebruik te maken van een gedeeld patiëntendossier. Naast actuele en juiste gegevens van de patiënt met betrekking tot diagnose, gewenste uitkomsten, afspraken over behandelingen en bereikte doelen, kunnen hierin het individueel behandelplan en zelfmanagementdoelen worden bijgehouden. |
| --- |

1.7 Geef aan wat bij u van toepassing is wat betreft het informatie- en communicatiesysteem.

| (kies het best passende antwoord) | **Ja** | **In**  **Ontwik-**  **keling** | **Nee** |
| --- | --- | --- | --- |
| Binnen de diabetespoli is één methode van registratie (standaardisatie van bijvoorbeeld zorguitkomsten) |  |  |  |
| Binnen de diabetespoli is één gezamenlijk patiëntendossier voor zorgverleners binnen de diabetespoli |  |  |  |
| Het patiëntendossier is ook toegankelijk voor zorgverleners in het ziekenhuis buiten de diabetespoli |  |  |  |
| Er zijn één of meerdere Keten Informatie Syste(e)m(en) (KIS) met zorgverleners buiten de diabetespoli |  |  |  |
| Heeft de diabetespoli toegang tot de informatie van huisartsen |  |  |  |
| In het patiëntendossier zijn voldoende mogelijkheden om het individuele zorgplan bij te houden |  |  |  |
| In het patiëntendossier zijn voldoende mogelijkheden om zelfmanagementdoelen bij te houden |  |  |  |
| Worden zorgverleners door het informatiesysteem (ZIS) eraan herinnerd de richtlijnen/ standaarden te volgen |  |  |  |
| Binnen de diabetespoli is één manier van rapportage voor alle zorgverleners |  |  |  |
| Binnen de diabetespoli is eenheid in verwijsformulieren |  |  |  |

1.8 Wordt er binnen uw diabetespoli gewerkt met een elektronisch patiëntendossier?

(kies het best passende antwoord)

- Ja, de diabetespoli werkt uitsluitend met een elektronisch patiëntendossier
- De diabetespoli werkt met een papieren patiëntendossier aangevuld met een elektronisch dossier voor bijvoorbeeld labuitslagen en briefwisselingen.

1.9 Welke zorgverleners hebben toegang tot het patiëntendossier?

| (kies het best passende antwoord) | **Alleen het papieren**  **dossier** | **Alleen het elektronisch**  **dossier** | **Beide** | **Geen toegang** |
| --- | --- | --- | --- | --- |
| Internist |  |  |  |  |
| Diabetesverpleegkundige |  |  |  |  |
| Cardioloog |  |  |  |  |
| Nefroloog |  |  |  |  |
| Oogarts |  |  |  |  |
| Optometrist |  |  |  |  |
| Apotheker |  |  |  |  |
| Diëtist |  |  |  |  |
| Podotherapeut |  |  |  |  |
| Psycholoog |  |  |  |  |
| Fysiotherapeut |  |  |  |  |
| Huisarts + POH |  |  |  |  |
| …………………… |  |  |  |  |

# 2. Multidisciplinaire samenwerking

| Multidisciplinaire samenwerking betekent dat verschillende disciplines met hun eigen expertise met elkaar samenwerken, waardoor kennis en zorg rondom een patiënt gebundeld wordt. Meestal worden multidisciplinaire richtlijnen of zorgprotocollen gebruikt. Samenwerkingsafspraken, verwijscriteria, verantwoordelijkheden en bevoegdheden dienen goed vastgelegd te zijn. |
| --- |

- 1. Met welke zorgverleners/instanties heeft uw diabetespoli schriftelijke werkafspraken gemaakt over het zorgprogramma diabetes

(meerdere antwoorden mogelijk)

- Internist
- Diabetesverpleegkundige (DVK)
- Huisarts + POH
- Diëtist
- Oogarts
- Optometrist
- Nefroloog
- Cardioloog
- Vaatchirurg
- Revalidatiearts
- Podotherapeut
- Psycholoog
- Apotheker
- Fysiotherapeut
- Verzorgingshuizen
- Verpleeghuizen
- Anders, namelijk ….

2.2 Wat doet de diabetespoli om multidisciplinaire samenwerking te faciliteren?

| (alle antwoorden aankruisen die van toepassing zijn) | **Ja** | **In ontwik-**  **keling** | **Nee** |
| --- | --- | --- | --- |
| De diabetespoli heeft protocollen over welke zorg door welke zorgverleners wordt verleend |  |  |  |
| Er zijn afspraken gemaakt over de medische verantwoordelijkheid bij taaksubstitutie (bijv. DVK neemt taken van de internist over) |  |  |  |
| De diabetespoli stimuleert het multidisciplinair overleg over diabetespatiënten |  |  |  |
| De diabetespoli heeft een gezamenlijk gedragen visie op scholing (doel en strategie) |  |  |  |
| De diabetespoli organiseert periodiek gezamenlijke scholing voor betrokken zorgverleners |  |  |  |
| Zorgverleners van verschillende disciplines houden gezamenlijk spreekuur |  |  |  |

- 1. Welke samenwerking vindt er plaats? Is deze samenwerking vastgelegd in een protocol? Wordt deze samenwerking geëvalueerd en worden de uitkomsten gebruikt om de kwaliteit van de samenwerking te verbeteren?

| (alle antwoorden aankruisen die van toepassing zijn) | Is er niet/ vindt niet plaats | Is nog niet in protocol vastgelegd | Is in protocol vast-  gelegd | Wordt periodiek geëva-  lueerd | Wordt gebruikt bij verbeter-acties |
| --- | --- | --- | --- | --- | --- |
| De diabetespoli heeft afspraken over samenwerking tussen de zorgverleners binnen het ziekenhuis |  |  |  |  |  |
| De diabetespoli heeft afspraken over overdracht van patiënten tussen de zorgverleners binnen het ziekenhuis |  |  |  |  |  |
| De diabetespoli heeft afspraken over overdracht van patiënten naar de zorgpartners buiten het ziekenhuis |  |  |  |  |  |
| De diabetespoli heeft multidisciplinaire afspraken over verwijs- en terugverwijscriteria (bv. naar diëtist en huisarts) |  |  |  |  |  |
| De diabetespoli heeft structureel multidisciplinair overleg over behandeling van patiënten |  |  |  |  |  |
| De diabetespoli heeft afspraken met andere specialisten bij multimorbiditeit |  |  |  |  |  |
| De diabetespoli organiseert regelmatig bijeenkomsten voor het bespreken van richtlijnen/ standaarden |  |  |  |  |  |
| De diabetespoli organiseert regelmatig bijeenkomsten voor het bespreken van taken en verantwoordelijkheden |  |  |  |  |  |
| De diabetespoli organiseert regelmatig bijeenkomsten voor het bespreken van problemen met betrekking tot de diabeteszorg |  |  |  |  |  |
| Anders, namelijk ……………… |  |  |  |  |  |

2.4 De consultatie functie van de internist ten behoeve van de 1^e^ lijn:

(kies het best passende antwoord)

- Wordt niet of te weinig gebruikt, omdat ……….
- Wordt volgens de afgesproken frequenties gebruikt
- Wordt te veel gebruikt, omdat …………..
- Er zijn geen afspraken over frequenties
- Gebruik is onbekend

2.5 Heeft de diabetespoli een multidisciplinair voetenteam?

(kies het best passende antwoord)

- Nee, er is geen multidisciplinair voetenteam
- Een multidisciplinair voetenteam is in ontwikkeling
- Ja, er is een multidisciplinair voetenteam, hierin participeren:
- Internist
- (Vaat)chirurg
- Diabetesverpleegkundige
- Podotherapeut
- Gipsverbandmeester
- Revalidatie arts
- Orthopedisch chirurg
- Orthopedisch schoenmaker,
- Anders, namelijk …

# 3. Patiëntgerichtheid

| Zelfmanagement: is erop gericht de patiënt in staat te stellen eigenmachtig te beslissen en handelen. Hiervoor moet de patiënt eerst inzicht krijgen in het ziektebeeld door adequate patiënteneducatie, inzage in de eigen medische gegevens en wordt er met de patiënt samen een individueel zorgplan opgesteld rekening houdend met diens wensen, mogelijkheden en omstandigheden. Dit individuele zorgplan wordt vervolgens samen met de patiënt regelmatig geëvalueerd en bijgesteld. |
| --- |

3.1 Hoe wordt zelfmanagement door de diabetespoli ondersteund?

(kruis aan wat van toepassing is)

- Wordt niet ondersteund door de diabetespoli
- Ondersteuning is momenteel in ontwikkeling
- Verspreiden van informatie (folders, boekjes)
- Cursussen voor zorgverleners
- Cursussen voor patiënten
- Anders, namelijk

3.2 Het opstellen van individuele zorgplannen

(kies het best passende antwoord)

- Wordt niet gestimuleerd door de diabetespoli
- Is in ontwikkeling binnen de diabetespoli
- Wordt actief door de diabetespoli gestimuleerd
- Wordt actief binnen de diabetespoli gestimuleerd en periodiek geëvalueerd aan de hand van vooraf bepaalde doelen

3.3 Het beleid van de diabetespoli ten aanzien van patiënteneducatie is:

(kies het best passende antwoord)

- Niet ontwikkeld binnen de diabetespoli
- In ontwikkeling binnen de diabetespoli
- Zoveel mogelijk tot een uniforme informatievoorziening te komen
- Zoveel mogelijk tot een uniforme informatievoorziening te komen en zorgverleners hier ook in te trainen

3.4 Hoe heeft de patiënt op inzage in zijn/haar medische gegevens?

(kies het best passende antwoord)

- De patiënt heeft alleen inzage in medische gegevens als hij/zij hier uitdrukkelijk om vraagt.
- Toegang tot medische gegevens is in ontwikkeling
- De patiënt kan de gegevens zien in de diabetespas
- De patiënt kan via een patiëntenportaal zijn gegevens inzien

3.5 Kan de patiënt zelf gegevens toevoegen aan zijn elektronische dossier?

(kies het best passende antwoord)

- Nee, er is geen elektronisch dossier
- Er is wel een elektronisch dossier, maar patiënt kan geen gegevens toevoegen
- De mogelijkheid voor de patiënt om zelf gegevens toe te voegen is in ontwikkeling
- Ja, via een patiëntenportaal

3.6 Patiëntenbelangen

| (kies het best passende antwoord) | Ja | In Ontwikkeling | Nee | Weet ik niet |
| --- | --- | --- | --- | --- |
| Is er in een protocol vastgelegd dat de patiënt geïnformeerd wordt over richtlijnen/standaarden (bv met de diabeteszorgwijzer)? |  |  |  |  |
| Is er bij de polikliniek een centrale plek (front-office) waar de patiënt met vragen terecht kan? (een balie, een centraal telefoonnummer of een website) |  |  |  |  |
| Worden spreekuurtijden van de verschillende hulpverleners op elkaar afgestemd? |  |  |  |  |
| Is er een vast aangewezen persoon tot wie de patiënt zich met al zijn vragen kan richten? |  |  |  |  |
| Wordt de privacy van de patiënt gegarandeerd bij een multidisciplinair zorgdossier? |  |  |  |  |
| Wordt de privacy van de patiënt gegarandeerd bij het verzamelen van gegevens voor bv spiegelinformatie? |  |  |  |  |

3.7 Hoe worden patiënten betrokken bij uw diabetespoli? Door middel van ….

(meerdere antwoorden zijn mogelijk)

- Patiënten zijn niet betrokken bij de diabetespoli
- Patiënten zijn nog niet structureel betrokken bij diabetespoli, maar we zijn dit wel aan het voorbereiden
- Cliëntenraad
- Klachtencommissie
- Structurele samenwerking met de regionale patiënten/ consumentenfederatie (NPCF of Zorgbelang)
- Structurele samenwerking met de patiëntenvereniging (DVN)
- Anders, namelijk.……

# 4. Resultaten

Dit aandachtsgebied bestaat uit de onderdelen registreren, verzamelen en verwerken van gegevens en de indicatoren.

| Gegevens worden geregistreerd op structuur-, proces- of uitkomstniveau. Uit de verzamelde data kunnen prestatie- of kwaliteitsindicatoren worden afgeleid om de kwaliteit van zorg te meten en verbeteren. Het is belangrijk hierbij ook patiënttevredenheidsindicatoren te gebruiken. |
| --- |

4.1 Worden er geregistreerde gegevens van zorgverleners verzameld voor feedback (spiegelinformatie/benchmark)?

(kies het best passende antwoord)

- Ja, ga door met vraag 4.2
- Nee, ga door met vraag 5.1

4.2 Hoe worden de geregistreerde gegevens van onderstaande zorgverleners verzameld voor feedback (spiegelinformatie/benchmark)?

| (Kies het best passende antwoord) | Via een elektronisch informatiesysteem | Via een papieren dossier | Worden niet verzameld |
| --- | --- | --- | --- |
| Internist |  |  |  |
| Diabetesverpleegkundige |  |  |  |
| Diëtist |  |  |  |
| Oogarts |  |  |  |
| Optometrist |  |  |  |
| Nefroloog |  |  |  |
| Cardioloog |  |  |  |
| Podotherapeut |  |  |  |
| Psycholoog |  |  |  |
| Fysiotherapeut |  |  |  |
| Anders. nl…. |  |  |  |

| Gegevens worden niet altijd juist ingevoerd of verwerkt. Een dataset kan door foutieve invoer onjuiste extreme waarden bevatten. Controle van gegevens is daarom van belang. |
| --- |

4.3 Hoe heeft de diabetespoli georganiseerd dat de door de zorgverleners geregistreerde en aangeleverde gegevens op juistheid worden gecontroleerd?

(meerdere antwoorden mogelijk)

- De diabetespoli heeft hier niets voor georganiseerd
- Dit gebeurt door de zorgverleners zelf
- De diabetespoli besteedt dit uit aan een onafhankelijke organisatie
- In het informatiesysteem zijn waarschuwingen ingebouwd om onjuiste data te voorkomen
- Anders, namelijk......

4.4 Wie bewerkt de aangeleverde resultaatgegevens van de zorgverleners tot feedbackgegevens/interne indicatoren?

(kies het best passende antwoord)

- De betreffende zorgverleners doen dit zelf
- De diabetespoli doet dit
- De diabetespoli besteedt dit uit aan een onafhankelijke organisatie
- Anders, namelijk......

4.5 Wie bewerkt de aangeleverde data van de zorgverleners tot externe verantwoordingsindicatoren aan bijvoorbeeld Zichtbare Zorg (ZIZO) of verzekeraars?

(kies het best passende antwoord)

- De betreffende zorgverleners doen dit zelf
- De diabetespoli doet dit
- De diabetespoli besteedt dit uit aan een onafhankelijke organisatie
- Anders, namelijk......

| Resultaatgegevens kunnen op verschillende manieren worden teruggekoppeld. Indien de diabetespoli of zorgverlener alleen een gemiddelde van een indicator gepresenteerd krijgt, is dat niet altijd voldoende aanleiding om hier verbeteracties op in te zetten. |
| --- |

4.6 Op welk niveau worden de gegevens geanalyseerd?

(Meerdere antwoorden mogelijk)

- Gegevens worden niet geanalyseerd
- Op patiëntniveau
- Op zorgverlener niveau
- Op niveau van de hele diabetespoli

4.7 Op welke wijze worden de gegevens geanalyseerd?

(Meerdere antwoorden mogelijk)

- Gegevens worden niet geanalyseerd
- Alleen gemiddelden worden bepaald
- Zowel gemiddelden als spreiding worden bepaald
- Ook individuele extreme waarden worden bepaald
- Op basis van medicatiegebruik worden subgroepen bepaald en op dat niveau wordt geanalyseerd
- Op basis van demografische gegevens worden subgroepen bepaald en op dat niveau wordt geanalyseerd
- Anders, namelijk …..

4.8 Welke dataset wordt op de diabetespoli geregistreerd?

(meerdere antwoorden mogelijk)

- Er worden geen indicatoren geregistreerd
- Basisset Prestatie-indicatoren Ziekenhuis 2005
- De Minimale Dataset (MDS) van de NZa
- De NDF e-Diabetes kernset
- De indicatorenset van de NHG
- Anders, namelijk …

4.9 Welke indicatorenset wordt berekend?

(Meerdere antwoorden mogelijk)

- ZIZO indicatoren
- “Kwaliteit van zorg in de etalage” indicatoren (NIV)
- Indicatoren voor verzekeraars
- NHG indicatoren
- Anders, nl. ……….

# 5. Kwaliteitsverbetering

Dit aandachtsgebied bestaat uit de onderdelen spiegelinformatie en benchmark, scholing en patiëntveiligheid.

5.1 Geef aan welke van onderstaande zaken uw organisatie meet ten behoeve van de kwaliteitsverbetering.

|  | **Niet**  **Gemeten** | **Wel gemeten.**  **Gebruikt voor kwa-liteitsverbetering?** | |
| --- | --- | --- | --- |
| (kies het best passende antwoord) |  | **ja** | **nee** |
| **Prestatie-indicatoren** |  |  |  |
| **Ervaringen van de patiënt:** bijvoorbeeld door afname van de CQ-index |  |  |  |
| **Meningen van verwijzers of andere partners in de diabeteszorg:** bijvoorbeeld tevredenheidonderzoeken of evaluatierapporten |  |  |  |
| **Klachtenregistratie:** de diabetespoli heeft een overzicht van alle klachten die met betrekking tot diabeteszorg binnenkomen |  |  |  |
| **Wachttijden bij zorgverleners*****:** zijn er wachtlijsten/wachttijden voor de toegang tot de zorgverleners die bij diabetes betrokken zijn? |  |  |  |
| * Zo ja: voor welke zorgverlener : ………………..  De wachttijd bedraagt gemiddeld: ……………….. |  |  |  |

# Spiegelinformatie en benchmark:

| Spiegelinformatie is het presenteren en vergelijken van resultaten of indicatoren binnen de organisatie om zodoende de zorg te verbeteren. |
| --- |

5.2 Met welke zorgverleners wordt de spiegelinformatie besproken?

(meerdere antwoorden mogelijk*)*

- Niet van toepassing. Er is geen spiegelinformatie aangeboden
- Internisten
- Diabetesverpleegkundigen
- Diëtisten
- Podotherapeuten
- Oogartsen
- Optometrist
- Cardiologen
- Nefrologen
- (Vaat)chirurgen
- Psychologen
- Fysiotherapeuten
- Apothekers
- Anders, namelijk………………………………………………………………………………

| Benchmarking: is het vergelijken van resultaten en indicatoren met andere organisaties met als doel de uitkomsten transparant te maken en de zorg te verbeteren |
| --- |

5.3 Wordt benchmarking bij de diabetespoli gebruikt om verbeteringen door te voeren:

(kies het best passende antwoord)

- Nee, benchmarking wordt niet gebruikt
- Benchmarking wordt incidenteel gebruikt
- Het beleid om benchmarking structureel in te zetten voor kwaliteitsverbetering is nog in ontwikkeling
- Benchmarking wordt structureel gebruikt om de kwaliteit te verbeteren

| Visitatie: is een vorm van intercollegiale toetsing gericht op het functioneren van een een individuele collega of een aantal samenwerkende zorgverleners. |
| --- |

5.4 Welke zorgverleners worden periodiek gevisiteerd?

(meerdere antwoorden mogelijk)

- Er vindt geen of slechts incidenteel visitatie plaats
- Internisten
- Diabetesverpleegkundigen
- Diëtisten
- Oogartsen
- Optometristen
- Cardiologen
- Nefrologen
- Anders, namelijk………………

# Scholing:

5.5 Hoe wordt het scholingsbeleid bepaald?

(kies het best passende antwoord)

- Scholing wordt niet gezien als een verantwoordelijkheid van de organisatie maar van de individuele zorgverlener
- Een scholingsbeleid binnen de diabetespoli is in ontwikkeling
- De diabetespoli kent een scholingsbeleid voor alle zorgverleners
- De diabetespoli kent een scholingsbeleid voor alle zorgverleners. Dit wordt regelmatig geëvalueerd en bijgesteld

5.6 Voor welke zorgverleners is in het afgelopen jaar bij- of nascholing georganiseerd?

(meerdere antwoorden mogelijk)

- Niet van toepassing, er is geen bij- of nascholing georganiseerd
- Internisten
- Diabetesverpleegkundigen
- Huisartsen
- POH-ers of doktersassistentes in de huisartsenpraktijk
- Diëtisten
- Oogartsen
- Optometristen
- Podotherapeuten
- Cardiologen
- Psychologen
- Anders, namelijk……………..

# Patiëntveiligheid:

5.7 Is er in een protocol vastgelegd hoe incidenten moeten worden gemeld?

(kies het best passende antwoord)

- Nee, dit is de verantwoordelijkheid van de individuele zorgverlener
- Nee, dit is in ontwikkeling
- Ja, incidenten worden aan de hoofdbehandelaar gemeld
- Ja, incidenten worden aan de hoofdbehandelaar en aan het ziekenhuis gemeld

| Patiëntveiligheid is de verantwoordelijkheid van elke individuele behandelaar. Om de zorgverlener hierbij te helpen, kunnen systemen gebruikt worden als “extra” beveiliging, die de behandelaar waarschuwen als er gevaarlijke situaties kunnen ontstaan. |
| --- |

5.8 Wordt er binnen de diabetespoli een systeem gebruikt dat systematisch scant en waarschuwt wanneer een patiënt mogelijke klinische schade kan gaan ondervinden?

(kies het best passende antwoord)

- Nee, dit wordt niet gebruikt
- Dit wordt door een aantal zorgverleners gebruikt
- Nee, dit is in ontwikkeling voor alle zorgverleners
- Ja, dit is voor alle zorgverleners operationeel

5.9 Hoe verloopt de bewaking van het medicatiedossier?

(kies het best passende antwoord)

- Via de medicijnpas van de patient
- Via het dossier van de huisarts
- Via de apotheek van de patient
- Via de ziekenhuisapotheek
- Er is geen bewaking geregeld

5.10 Worden er binnen uw diabetespoli subgroepen onderscheiden om gericht beleid in te kunnen zetten (bijvoorbeeld voor mensen met nierproblemen)?

(Kies het best passende antwoord)

- Nee, dit onderscheid wordt niet gemaakt
- Hier wordt incidenteel naar gekeken
- Op de diabetespoli worden structureel subgroepen van patiënten onderscheiden
- Hier wordt structureel naar gekeken en waar mogelijk ook beleid op ingezet

5.11 Wordt er binnen uw diabetespoli speciaal beleid ingezet om moeilijk bereikbare patiëntgroepen de juiste zorg te geven?

(Meerdere antwoorden mogelijk)

- Nee, dit vindt niet plaats
- Ja, voor mensen met een lage sociaal economische status
- Ja, voor mensen van allochtone afkomst
- Ja, voor zorgmijders
- Ja, voor minder mobiele mensen
- Ja, voor mensen die veel verschillende medicijnen gebruiken (polyfarmacie)
- Ja, voor mensen met multimorbiditeit
- Ja, voor mensen die meerdere specialisten bezoeken
- Anders, namelijk …

# 6. Kwaliteitsbeleid

Dit aandachtsgebied bestaat uit de onderdelen structureel kwaliteitsbeleid, kwaliteitssysteem en kwaliteitsdocumenten.

# Structureel kwaliteitsbeleid

6.1 Wie is de voortrekker van het kwaliteitsmanagementbeleid ten aanzien van diabetes in uw polikliniek?

(kies het best passende antwoord)

- Een stuurgroep of commissie
- De kwaliteitsfunctionaris
- Een extern bedrijf of adviseur
- Medische staf
- De directie of het management van het ziekenhuis
- Het hoofd van de polikliniek
- Alle internisten samen
- De internist die het meest gespecialiseerd is in diabetes
- Alle in diabetes gespecialiseerde internisten samen
- Anders, namelijk …..

6.2 Op welke manier is het kwaliteitsbeleid structureel ingebed in uw organisatie?

(meerdere antwoorden mogelijk)

- Het is niet structureel ingebed
- Er is een speciaal intern budget gereserveerd voor kwaliteitsbeleid
- Er zijn één of meer stuurgroepen (of commissies) gevormd
- Er zijn één of meer kwaliteitsfunctionarissen aangesteld
- Er is een extern bedrijf of adviseur betrokken
- Anders, namelijk………………………………..

| Cyclisch kwaliteitsbeleid wordt vaak gebruikt als middel om voortdurend kwaliteit te verbeteren. Een bekend voorbeeld hiervan is de kwaliteitscirkel van Deming, waarbij de stappen plan-do-check-act (PDCA) worden doorlopen. |
| --- |

6.3 Wordt binnen de diabetespoli een vorm van cyclisch kwaliteitsbeleid gebruikt als instrument om de kwaliteit van diabeteszorg te verbeteren?

(kies het best passende antwoord)

- Nee
- Ja, maar incidenteel
- Ja, nu nog incidenteel, maar structureel gebruik is in ontwikkeling
- Ja, we maken daar structureel gebruik van

# kwaliteitssysteem

6.4 Werkt uw organisatie met een gecertificeerd kwaliteitssysteem?

(kies het best passende antwoord)

- Ja, namelijk:
- ISO
- INK
- HKZ
- NIAZ
- Anders, namelijk……………
- Nee

# kwaliteitsdocumenten

6.5 Kunt u aangeven welke van de volgende documenten uw diabetespoli heeft?

| (kies het best passende antwoord) | **Ja** | **In**  **ontwikkeling** | **Nee** |
| --- | --- | --- | --- |
| **Missiedocument:** visie en prioriteiten van de organisatie |  |  |  |
| **Kwaliteitsactieplan voor de organisatie:** maatregelen voor de implementatie en de planning van acties om kwaliteitsdoelen te bereiken |  |  |  |
| **Jaarlijks kwaliteitsrapport:** rapportage over alle uitgevoerde activiteiten om kwaliteit te borgen en de resultaten daarvan |  |  |  |
| **Kwaliteitshandboek:** beschrijving van alle procedures die de organisatie gebruikt voor kwaliteitsborging en de verantwoordelijke personen daarvoor |  |  |  |
| Is het **kwaliteitshandboek** beschikbaar voor alle medewerkers binnen de organisatie? |  |  |  |

6.6 Welke uitspraak is voor de diabetespoli het meest van toepassing?

(meerdere antwoorden mogelijk)

- Onze afdeling maakt gebruik van het kwaliteitsdocumenten van het hele ziekenhuis
- Onze afdeling maakt gebruik van het kwaliteitsdocumenten van afdeling interne
- De diabetespoli heeft eigen kwaliteitsdocumenten
- De diabetespoli heeft geen kwaliteitsdocumenten

# Toekomst en slotvragen

Wat zijn de twee belangrijkste prioriteiten in kwaliteitsmanagementbeleid voor het volgende jaar?

(2 antwoorden aankruisen)

- (Verder) formuleren van procedures
- (Verder) formuleren van protocollen
- Kwaliteits(verbeter)projecten aan de hand van spiegelinformatie
- Kwaliteits(verbeter)projecten aan de hand van benchmarking
- Consequent volgen van de kwaliteitscirkel (plan-do-check-act)
- Patiëntervaringen-onderzoeken
- Visitering van een aantal beroepsgroepen binnen de zorggroep
- Accreditatie van een aantal beroepsgroepen binnen de zorggroep
- Certificering van een aantal beroepsgroepen binnen de zorggroep
- Accreditatie of certificering van de diabetespoli als geheel
- Verbeteren van ICT mogelijkheden
- Introduceren/verbeteren van mogelijkheden tot zelfmanagement van patiënten
- Competenties en scholing van de zorgverleners op peil brengen/houden
- Verbeteren van de verwijzing/samenwerking met zorgverleners buiten de organisatie
- Anders, namelijk …..

Heeft u nog vragen of opmerkingen over de vragenlijst of anderszins? Dan kunt u deze hieronder invullen.

.......................................................................................................................................................................................................................................................................................................

Wie heeft de vragenlijst ingevuld?

- Directeur
- Manager
- Kwaliteitsfunctionaris
- Internist met specialisatie diabetes
- Kaderarts
- Afdelingshoofd
- Anders, namelijk ………

Toolbox

Vanuit het project “Aan de slag met kwaliteitsbeleid” willen we u, als deelnemer, graag een online toolbox aanbieden, die instrumenten en praktijkcases bevat, die nu al door organisaties succesvol worden ingezet op het gebied van kwaliteit. Deze instrumenten worden momenteel door ons verzameld, waarbij wij ook uw hulp nodig hebben.

We willen u daarom vragen of u de door u gebruikte kwaliteitsinstrumenten beschikbaar wilt stellen om te plaatsen in deze toolbox. Daarbij valt te denken aan documenten, draaiboeken, folders e.d. die andere organisaties kunnen inspireren het eigen kwaliteitsbeleid te optimaliseren. Bij vermelding van uw tools wordt uiteraard zorgvuldig omgegaan met bronvermelding.

U kunt hieronder aangeven of u instrumenten ter beschikking heeft.

- Ja, ik heb tools beschikbaar op het gebied van kwaliteitsmanagementbeleid diabetes. U kunt hiervoor contact met mij opnemen.
- Ik weet op dit moment niet of ik tools beschikbaar heb. Ik kom hier mogelijk later op terug
- Nee, ik heb (nog) geen tools beschikbaar voor dit project.

**Hartelijk dank voor het invullen!**

In verband met het wetenschappelijk onderzoek worden de resultaten van de enquête gelijktijdig aan alle respondenten teruggekoppeld. Uw resultaten worden

begin maart 2012 per mail toegezonden in de vorm van een spinnenwebgrafiek.

U krijgt dan ook een toegangscode tot de online toolbox op www.diabeteskwaliteitsbeleid.nl.

Na ongeveer 12 maanden ontvangt u nogmaals een uitnodiging voor het invullen van de enquête. Ook van die resultaten ontvangt u weer een spinnenwebgrafiek, zodat u de verandering in uw kwaliteitsmanagementbeleid kunt zien.

Heeft u in de tussentijd vragen of wenst u meer informatie, kijkt u dan op www.diabeteskwaliteitsbeleid.nl of neem contact op met Marjo Campmans

(onderzoek@diabeteskwaliteitsbeleid.nl of tel: 06-23295934)

Dank u voor uw deelname.

Projectteam ‘Aan de slag met kwaliteitsbeleid’
