# Supplementary material for: Diabetes quality management in Dutch care groups and outpatient clinics: a cross-sectional study
Source: BMC Res Notes. 2014 Aug 7;7:497. doi: 10.1186/1756-0500-7-497 (PMC4132241; doi:10.1186/1756-0500-7-497)
Supplement: Additional file 3 — Scoring of questionnaire for diabetes care groups. [file 1756-0500-7-497-S3.pdf]

CARE GROUPS

Legend

orange: Weighting factor
red: special scoring (maximised)
**Achieved score of this outpatient clinic**
**Maximum possible score**

1. Organisation of care

1.1 The diabetes care program …

#	(choose the best possible answer)	No	Yes	Under development		score	max score
		0.000	0.167	0.083			
1	Is with respect to the protocol recorded in writing		x			0.17	0.17
2	Its contents is based on evidence-based standards and guidelines		x			0.17	0.17
3	Is with regard to the agreements on organisation and implementation recorded in writing			x		0.08	0.17
4	Includes a description of the functions of all the different care providers		x			0.17	0.17
5	Is available in writing for all care providers involved			x	*)	0.08	0.17
6	Is digitally available for all care providers involved			x	*)		
7	Is structurally maintained and updated	x				0.00	0.17
	*) The maximum score of sub question 5 and 6 counts					**0.67**	**1.00**

1.2 Who are involved in the designing, implementing and/or evaluation of the diabetes care program?

#	(Please tick all that apply, more answers possible)	designing	implementing	evaluating and adapting	Not involved	score	max score
		0.042	0.083	0.125	0.000		
1	The management	x				0.04	0.13
2	A general practitioners with specialty in diabetes care		x	x		0.13	0.13
3	A steering committee or commission		x			0.08	0.13
4	A quality officer			x		0.13	0.13
5	An external company or consultant		x			0.08	0.13
6	(Representative(s) (of)) health care providers	x				0.04	0.13
7	Representative(s) of patients	x				0.04	0.13
8	Other, namely …..	x				0.04	0.13
	horizontal maximum score counts					**0.58**	**1.00**

1.3 How often is the care program being evaluated?

#	(choose the best possible answer)			weight	score	max score
1	Not applicable. The care program is not being evaluated			0.000	0.00	0.00
2	The care program is not being evaluated with a regular frequency	x		0.500	0.50	0.50
3	Annually			1.000	0.00	1.00
4	Biannually			1.000	0.00	1.00
5	Otherwise, namely		namely……………	0.500	0.00	0.50
	total score maximised on 1 point				**0.50**	**1.00**

1.4 The content of the care program is adjusted if:

#	(several answers possible)			weight	score	max score
1	Not applicable. The content of the care program is being not adjusted	x		0.000	0.00	0.00
2	The standard of the Dutch General Practitioners is adjusted	x	*)	0.500	0.50	0.50
3	The care standard of the Dutch Diabetes Federation (NDF) is revised	x	*)	0.500	0.50	0.50
4	New evidence-based guidelines are available		*)	0.500	0.00	0.50
5	There are new medications coming on the market	x	**)	0.500	0.50	0.50
6	Content of bundled payment contracts has changed	x	**)	0.500	0.50	0.50
7	Outcomes / results of care give cause	x	**)	0.500	0.50	0.50
8	Otherwise, namely		namely: ……………………………..	0.000	0.00	0.00
	*) The sum of sub questions 2, 3 and 4 is maximised on 0,5 point				**1.00**	**1.00**
	**) The sum of sub questions 5, 6 and 7 is maximised on 0,5 point					

1.5 Does the care group have a policy on coordination of care?

#	(choose the best possible answer)		weight	score	max score
1	The care group does not manage on coordination; practices themselves determine how care is coordinated now and in future.		0.000	0.00	0.00
2	Coordination of care is not yet a centrally planned activity of the care group, but is planning to do so in future.	x	0.500	0.50	0.50

	3	The care group stimulates one care coordinator per general practice.					1.000	0.00	1.00
	4	The care group itself has a care coordinator who supports care coordinators in practices.					1.000	0.00	1.00
								**0.50**	**1.00**
**1.6**	To your opinion, do patients within your care group receive uniform information and advice from the various health care providers about diabetes?								
		(choose the best possible answer)							
	1	Yes					1.000	0.00	1.00
	2	Partly	x				0.500	0.50	0.50
	3	No					0.000	0.00	0.00
	4	I do not know					0.000	0.00	0.00
								**0.50**	**1.00**
**1.7**	To what extend does the care group have information on patients who enter of exit the care program?								
		(choose the best possible answer)							
	1	The care group has no view of the inflow and outflow of patients					0.000	0.00	0.00
	2	The care group only has a view of the influx of patients	x				0.500	0.50	0.50
	3	The care group has a view of both the in- and outflow of patients					1.000	0.00	1.00
	4	The care group only has a view of the outflow of patients					0.500	0.00	0.50
								**0.50**	**1.00**
**1.8**	To what extent does the care group have view on the reasons for outflow of patients?								
		(several answers possible)							
	1	Not applicable. The care group has no view on the outflow of patients.					0.000	0.00	0.00
	2	The care group has no insight into the reasons for outflow.					0.250	0.00	0.25
	3	The care group knows who are deceased	x				0.250	0.25	0.25
	4	The care group knows who moved					0.250	0.00	0.25
	5	The care groups knows who were transferred to secondary care.	x				0.250	0.25	0.25
								**0.50**	**1.00**
**1.9**	Some patients do not or very irregularly come to the regular checks and thus do not receive the care which was agreed in the care protocol within the care group.								
			**No**	**Yes**	**Do not know**	**Under development**			
		(choose the best possible answer)	0.000	0.250	0.000	0.125			
	1	Do practices have insight into this?				x		0.13	0.25
	2	Does the care group have insight into this?		x				0.25	0.25
	3	Does the care group take action on this?			x			0.00	0.25
	4	Do practices take action on this?		x				0.25	0.25
								**0.63**	**1.00**
**1.10**	To what extent is a call system for patients being used within the care group?								
		(choose the best possible answer)							
	1	The care group has no central call system and leaves calling patients entirely to individual practices					0.000	0.00	0.00
	2	The care group promotes the use of a call system in practices	x				0.333	0.33	0.33
	3	A call system for patients is compulsory for all individual practices within the care group					0.667	0.00	0.67
	4	The care group has a central call system that gives practitioners the ability to centrally call their patients for annual monitoring.					1.000	0.00	1.00
								**0.33**	**1.00**
**1.11**	At this moment, within the care group:								
		(choose the best possible answer)							
	1	Different practices use different general practitioners' (GP) information systems					0.000	0.00	0.00
	2	Both GP and chain information system are used alongside					0.333	0.00	0.33
	3	All practices use the same GP information system	x				1.000	1.00	1.00

4	All practices use the same chain information system					1.000	0.00	1.00
5	Is strived for to start using the same GP information system					0.500	0.00	0.50
6	Is strived for to start using the same chain information system					0.500	0.00	0.50
7	Otherwise, namely		namely……………			0.500	0.00	0.50
							1.00	1.00

1.12 Information and communication system

		Yes	Under development	No		
	(choose the best possible answer)	0.143	0.071	0.000		
1	Within the care group is one method of recording (for example, standardisation of care outcomes)	x			0.14	0.14
2	An individual treatment plan can be registered in the GP or chain information system		x		0.07	0.14
3	An individual care plan can be registered in the GP or chain information system		x		0.07	0.14
4	Individual self-management targets can be registered in the GP or chain information system			x	0.00	0.14
5	Are care providers being reminded to follow protocol by the GP or chain information system		x		0.07	0.14
6	There is unity in referral forms within the care group	x			0.14	0.14
7	There is unity in laboratory forms within the care group	x			0.14	0.14
					**0.64**	**1.00**

1.13 If the care group uses a chain information system, which care providers have access to the (electronic) medical record?

		Yes	No	Not applicable / is not employed by the care group			
	(choose the best possible answer)	0.200	0.000	0.000			
1	General Practitioner	x				0.20	0.20
2	Practice nurse		x			0.00	0.20
3	Diabetes nurse			x		0.00	0.20
4	Endocrinologist		x			0.00	0.20
5	Ophthalmologist	x				0.20	0.20
6	Optometrist	x				0.20	0.20
7	Pharmacist	x				0.20	0.20
8	Dietician	x				0.20	0.20
9	Podiatrist	x				0.20	0.20
10	Physiotherapist	x				0.20	0.20
11	Other, namely	x			namely……………	0.20	0.20
12	Other, namely	x			namely……………	0.20	0.20
			5 care providers gives maximum of 1 point			**1.00**	**1.00**

	Question number	score achieved	maximum points	achieved score	experts' weighting	achieved weighted score	weighted maximum score
1. Care program	1.1-1.4	2.75	4	69%	35%	24%	31%
2. Continuity and coordination	1.5-1.10	2.96	6	49%	35%	17%	46%
3. Communication and information	1.11-1.13	2.64	3	88%	30%	26%	23%
**TOTAL SCORE Organisation of care**		**8.35**	**13**	64%	100%	68%	100%

2. Multidisciplinary teamwork

2.1 Which care providers have written work agreements concerning the diabetes care program within the care group (NB. This eliminates the need to have a contract)

		yes	Under development	No		
	(Tick the appropriate box)	0.100	0.050	0.000		
1	General practitioner and practice nurse	x			0.10	0.10
2	Diabetes nurse	x			0.10	0.10
3	Endocrinologist	x			0.10	0.10
4	Ophthalmologist	x			0.10	0.10
5	Optometrist	x			0.10	0.10
6	Pharmacist	x			0.10	0.10
7	Dietician		x		0.05	0.10
8	Podiatrist	x			0.10	0.10
9	Psychologist	x			0.10	0.10
10	Physiotherapist	x			0.10	0.10
11	Other, namely			x	0.00	0.10
			10 care providers gives maximum score of 1 point		**0.95**	**1.00**

2.2 How does the care group facilitate multidisciplinary teamwork?

		Yes	Under development	No		
	(choose the best possible answer)	0.250	0.125	0.000		
1	There is an explicit description of the roles and responsibilities of health professionals involved	x			0.25	0.25
2	Arrangements have been made about job substitution (e.g. practice nurses taken over GPs' tasks)		x		0.13	0.25
3	The care group promotes multidisciplinary consultation on diabetes patients (structural consultation between at least two collaborating care providers with different expertise)			x	0.00	0.25
4	The care group periodically organises joint training for care providers involved		x		0.13	0.25
					**0.50**	**1.00**

2.3 What kind of cooperation is there in your care group? Has this been defined in a protocol? Is this cooperation being evaluated and are the results of this evaluation being used to improve the quality of the cooperation?

		Do not exist/does not take place	Are defined in protocol	Are periodically evaluated	Are used in improvement efforts			
	(choose the best possible answer)	0.000	0.048	0.095	0.143			
1	The care group has agreements on cooperation between care providers	x					0.00	0.14
2	The care group has agreements on transfer of patients between health care providers <u>within</u> the care group		x				0.05	0.14
3	The care group has agreements on transfer of patients to health care providers <u>outside</u> of the care group				x		0.14	0.14
4	The care group has agreed on multidisciplinary referral and back referral criteria (e.g. between dietician and endocrinologist)		x				0.05	0.14
5	The care group has ongoing consultations with health care providers about the outcomes of care (e.g., feedback meetings)		x				0.05	0.14
6	The care group organises regular meetings to discuss guidelines / standards			x			0.10	0.14
7	The care group organises regular meetings to discuss roles and responsibilities			x			0.10	0.14
8	Otherwise, namely ………………	x				………….	0.00	0.14
							**0.48**	**1.00**

2.4 The consultation function of the endocrinologist:

	(several answers possible)				
1	Is recorded in the protocol of the care program		0.200	0.00	0.20
2	Arrangements have been made on the criteria of consultation		0.200	0.00	0.20
3	There are agreements on the frequency of consultation		0.200	0.00	0.20
4	It states that consultation takes place:	x	0.200	0.20	0.20
4-1	via telephone consultation	x	0.000	0.00	0.00
4-2	via e-mail consultation	x	0.000	0.00	0.00
5	Appointments are structurally evaluated and adjusted if necessary	x	0.200	0.20	0.20
				**0.40**	**1.00**

	Question number	score achieved	maximum points	achieved score	experts' weighting	achieved weighted score	weighted maximum score
Work agreement	2.1; 2.3.5; 2.4	1.40	2.14	65%	30%	20%	54%
Tasks and responsibilities	2.2.1; 2.2.2; 2.3.7	0.47	0.64	73%	20%	15%	16%
Teamwork/consultation/shared education/guidelines	2.3.1;2.2.3; 2.2.4; 2.3.6; 2.3.8	0.22	0.93	24%	30%	7%	23%
Transfer and referral	2.3.2; 2.3.3; 2.3.4	0.24	0.43	56%	20%	11%	11%
**TOTAL SCORE Multidisciplinary teamwork**		**2.33**	**4.00**	58%	100%	52%	100%

<u>3. Patient centeredness</u>

3.1 How is self-management in diabetic patients supported by the care group?

		(choose the best possible answer)							
	1	Does not take place					0.000	0.00	0.00
	2	Support is currently under development					0.250	0.00	0.25
	3	Dissemination of information (brochures, booklets)	x				0.500	0.50	0.50
	4	Courses for care providers					1.000	0.00	1.00
	5	Courses for care patients					1.000	0.00	1.00
	6	Otherwise, namely		namely……………			0.250	0.00	0.25
								**0.50**	**1.00**
**3.2**	The use of individual care plans:								
		(choose the best possible answer)							
	1	Is not stimulated by the care group					0.000	0.00	0.00
	2	Is under development within the care group					0.333	0.00	0.33
	3	Is actively encouraged by the care group					0.667	0.00	0.67
	4	Is actively encouraged within the care group and periodically evaluated on the basis of predetermined goals	x				1.000	1.00	1.00
								**1.00**	**1.00**
**3.3**	The policy of the care group regarding patient education is:								
		(choose the best possible answer)							
	1	Not developed within the care group					0.000	0.00	0.00
	2	In development within the care group					0.250	0.00	0.25
	3	That practice self-regulate the patient education	x				0.500	0.50	0.50
	4	To achieve a uniform information as possible by means of coordination					0.750	0.00	0.75
	5	To achieve a uniform information as possible by means of coordination and also to train care givers in uniform information					1.000	0.00	1.00
								**0.50**	**1.00**
**3.4**	Does the patient have access to her/his medical records?								
		(choose the best possible answer)							
	1	Is not stimulated by the care group					0.000	0.00	0.00
	2	Is under development within the care group					0.333	0.00	0.33
	3	Is actively encouraged by the care group	x				0.667	0.67	0.67
	4	Is actively encouraged within the care group and periodically evaluated on the basis of predetermined goals					1.000	0.00	1.00
	5	Otherwise, namely		namely……………			0.333	0.00	0.33
								**0.67**	**1.00**
**3.5**	Can the patient data add to her/his electronic records?								
		(choose the best possible answer)							
	1	No, there is no electronic record					0.000	0.00	0.00
	2	There is an electronic file, but patient can not add data					0.333	0.00	0.33
	3	The ability of the patient to add data itself is under development					0.667	0.00	0.67
	4	Yes, through a patient portal	x				1.000	1.00	1.00
								**1.00**	**1.00**
**3.6**	Patient interests								
			**Yes**	**Under development**	**No**				
		(choose the best possible answer)	0.143	0.071	0.000				
	1	Is there an established protocol that the patient is informed of guidelines / standards (e.g. "de diabetes zorgwijzer)	x					0.14	0.14
	2	Does the website of the care group inform patients about the care group?		x				0.07	0.14
	3	Is there a central location in the care group (front-office) where the patient can ask questions? (a desk, a central phone number or a website)		x				0.07	0.14
	4	Are consultation hours are from different care providers coordinated and tuned?			x			0.00	0.14
	5	Is there a fixed designated person to whom the patient can address his/her questions?		x				0.07	0.14
	6	Is the patient privacy guaranteed in a multidisciplinary care record?			x			0.00	0.14
	7	Is the privacy of the patient guaranteed in the collection of data, for example for a benchmark?		x				0.07	0.14
								**0.43**	**1.00**
**3.7**	How are patients involved in your care group? By means of								

	(several answers possible)					
1	Client-board at care group level	x		0.250	0.25	0.25
2	A complaints committee at care group level	x		0.250	0.25	0.25
3	Structural cooperation with regional patient / consumer federation	x		0.250	0.25	0.25
4	Structural cooperation with the national patient organisation (Dutch diabetes cooperation)	x		0.250	0.25	0.25
5	Patients are not structurally involved in the care group, but we are preparing to do so.	x		0.130	0.13	0.13
6	Patients are not involved in the care group	x		0.000	0.00	0.00
7	Otherwise, namely		namely……………	0.130	0.00	0.13
			Total score maximised on 1 point		**1.00**	**1.00**

	Question number	score achieved	maximum points	achieved score	experts' weighting	achieved weighted score	weighted maximum score
Self-management	3.1	0.50	1	50%	20%	10%	14%
Individual care plan	3.2	1.00	1	100%	20%	20%	14%
Policy on patient education	3.3	0.50	1	50%	20%	10%	14%
Inspection of medical file	3.4 and 3.5	1.67	2	83%	12%	10%	29%
Patient interests	3.6	0.43	1	43%	18%	8%	14%
Patient involvement	3.7	1.00	1	100%	10%	10%	14%
**TOTAL SCORE Patient centeredness**		**5.10**	**7**	73%	100%	68%	100%

4. Performance management

4.1 How are the records of the following care providers collected for the care group?

		**Yes**	**Under development**	**No**			
	(choose the best possible answer)	0.125	0.063	0.000			
1	General practitioner and practice nurse	x				0.13	0.13
2	Diabetes nurse		x			0.06	0.13
3	Endocrinologist			x		0.00	0.13
4	Ophthalmologist		x			0.06	0.13
5	Optometrist			x		0.00	0.13
6	Dietician			x		0.00	0.13
7	Podiatrist			x		0.00	0.13
8	Physiotherapist			x		0.00	0.13
9	Other, namely			x	namely……………	0.00	0.13
			8 care providers gives the maximum score of 1 point			**0.25**	**1.00**

4.2 How has the care group organised the checking of correctness of the data recorded and supplied by care providers?

	(several answers are possible)					
1	The care group has not organised anything for this			0.000	0.00	0.00
2	This is done by the caregivers themselves			0.200	0.00	0.20
3	The care group outsources this to an independent organisation			0.400	0.00	0.40
4	In the information system has integrated alerts to prevent erroneous data	x		0.400	0.40	0.40
5	Otherwise, namely		namely……………	0.200	0.00	0.20
			Total score maximised on 1 point		0.40	**1.00**

4.3 Who edits the submitted results data from the providers to feedback data / **internal** indicators?

	(choose the best possible answer)					
1	The respective care providers do this themselves			0.333	0.00	0.33
2	The care group does this			0.667	0.00	0.67
3	The care group outsources this out to an independent organisation	x		1.000	1.00	1.00
4	Otherwise, namely		namely ……………	0.333	0.00	0.33
					**1.00**	**1.00**

4.4 Who processes the data supplied from the providers to **external** accountability indicators, for example Visible Care (ZIZO) or insurers?

	(choose the best possible answer)					
1	The respective providers do this themselves			0.333	0.00	0.33
2	The care group does this			0.667	0.00	0.67
3	The care group outsources this out to an independent organisation	x		1.000	1.00	1.00
4	Otherwise, namely		namely ……………	0.333	0.00	0.33
					**1.00**	**1.00**

4.5 At what level are the data analysed?

	(several answers possible)					
1	Data are not analysed			0.000	0.00	0.00

No.	Answer	Answer given				Weighting	Achieved score	Maximum score
2	At the patient level					0.250	0.00	0.25
3	At the practice level	x				0.250	0.25	0.25
4	On GP group (Hagro) level					0.250	0.00	0.25
5	At the care group level	x				0.250	0.25	0.25
							0.50	1.00

4.6 How are the data analysed?

No.	(several answers possible)	Answer given				Weighting	Achieved score	Maximum score
1	Data are not analysed					0.000	0.00	0.00
2	Only averages are determined					0.200	0.00	0.20
3	Both averages and dispersion are determined	x				0.200	0.20	0.20
4	Also individual extreme values are determined					0.200	0.00	0.20
5	On the basis of the use of medication sub-groups will be determined, and data are analysed at that level					0.200	0.00	0.20
6	Based on demographic data subsets are determined and data are analysed at that level	x				0.200	0.20	0.20
7	Other, namely		namely……………			0.200	0.00	0.20
				Total score maximised on 1 point			0.40	1.00

4.7 Which dataset is recorded in the care group?

No.	(several answers possible)	Answer given				Weighting	Achieved score	Maximum score
1	There is no specific data set recorded					0.000	0.00	0.00
2	The minimum data set (MDS) of the Dutch Healthcare Authority (Nza), Visible Care					1.000	0.00	1.00
3	The e-Diabetes core set of the Dutch Diabetes Federation (NDF)	x				1.000	1.00	1.00
4	Other, namely		namely……………			1.000	0.00	1.00
			If a specific data set is recorded, the score becomes 1 point				1.00	1.00

4.8 Which indicators are calculated?

No.	(several answers possible)	Answer given				Weighting	Achieved score	Maximum score
1	Visible care (ZIZO) indicators					1.000	0.00	1.00
2	Indicators for insurers	x				1.000	1.00	1.00
3	Indicators of the Dutch General Practitioners (NHG)	x				1.000	1.00	1.00
4	Other, namely		namely……………			1.000	0.00	1.00
			If any indicators are used, the score becomes 1 point				1.00	1.00

	Question number	score achieved	maximum points	achieved score	experts' weighting	achieved weighted score	weighted maximum score
Registering results	4.1	0.25	1	25%	30%	8%	13%
Control of results	4.2	0.40	1	40%	10%	4%	13%
Processing of results	4.3 and 4.4	2.00	2	100%	10%	10%	25%
Analysing results	4.5 and 4.6	0.90	2	45%	20%	9%	25%
Measured outcomes	4.7 and 4.8	2.00	2	100%	30%	30%	25%
**TOTAL SCORE Performance management**		5.55	8	69%	100%	61%	100%

5. Quality improvement policy

5.1 What results does your organisation use for quality improvements?

No.	(choose the best possible answer)	Not measured	Measured! Used for quality improvement? Yes	Measured! Used for quality improvement? No		Achieved score	Maximum score
		0.000	0.200	0.100			
1	Performance indicators	x				0.00	0.20
2	Experiences of the patient: for example, by asking patients to complete the CQ index		x			0.20	0.20
3	Views of the referrers or other partners in sequenced care : e.g. satisfaction surveys or evaluation			x		0.10	0.20
4	Joint complaints registration: the care group has an overview of all complaints that are received by the care group		x			0.20	0.20
5	Waiting times for care providers: are waiting lists / waiting times measured for access to health care within the organisation			x		0.10	0.20
6	Otherwise, namely	x			namely……………	0.00	0.20
						0.60	1.00

5.2 With which care providers is the mirror information discussed?

No.	(several answers possible)	Answer given				Weighting	Achieved score	Maximum score
1	Not applicable. There is no mirror information offered					0.000	0.00	0.00
2	General practitioners					0.200	0.00	0.20
3	Practice nurses	x				0.200	0.20	0.20

	4	Diabetes nurses	x		0.200	0.20	0.20
	5	Dieticians	x		0.200	0.20	0.20
	6	Podiatrists	x		0.200	0.20	0.20
	7	Ophthalmologists	x		0.200	0.20	0.20
	8	Optometrists	x		0.200	0.20	0.20
	9	Endocrinologists			0.200	0.00	0.20
	10	Psychologists			0.200	0.00	0.20
	11	Other, namely		namely…………..	0.200	0.00	0.20
				With 5 care providers, the maximum score is reached		**1.00**	**1.00**
**5.3**	Is benchmarking being used in your care group to make improvements?						
		(choose the best possible answer)					
	1	No, benchmarking is not being used			0.000	0.00	0.00
	2	Benchmarking is occasionally used			0.333	0.00	0.33
	3	The policy to structurally use benchmarking for quality improvement is still under development			0.667	0.00	0.67
	4	Benchmarking is structurally used for quality improvement	x		1.000	1.00	1.00
						**1.00**	**1.00**
**5.4**	On what basis is a care provider inspected within the care group?						
		(choose the best possible answer)					
	1	Care providers are not being inspected by the care group			0.000	0.00	0.00
	2	Newly acceded providers are inspected			0.333	0.00	0.33
	3	Caregivers who score poorly on the outcomes of care are inspected			0.667	0.00	0.67
	4	All caregivers are periodically inspected by the care group	x		1.000	1.00	1.00
						**1.00**	**1.00**
**5.5**	Which care providers within the care group are periodically inspected?						
		(several answers possible)					
	1	There is no or only occasional inspection			0.000	0.00	0.00
	2	General practitioners/General Practices			0.200	0.00	0.20
	3	Dieticians	x		0.200	0.20	0.20
	4	Ophthalmologists	x		0.200	0.20	0.20
	5	Endocrinologists	x		0.200	0.20	0.20
	6	Podiatrists	x		0.200	0.20	0.20
	7	Other, namely………………		namely…………..	0.200	0.00	0.20
						**0.80**	**1.00**
**5.6**	How is the training policy designed?						
		(choose the best possible answer)					
	1	Not applicable: training is not regarded as a responsibility of the care group, but as a responsibility of individual care providers			0.000	0.00	0.00
	2	The training policy within the care group is under development			0.250	0.00	0.25
	3	The care group has a training policy for part of the care providers within the care group			0.500	0.00	0.50
	4	The care group has a training policy for all care providers within the care group			0.750	0.00	0.75
	5	The care group has a training policy for all care providers within the care group, which is regularly evaluated and updated	x		1.000	1.00	1.00
						**1.00**	**1.00**
**5.7**	For which care providers has the care group organised continuing education in the past year?						
		(several answers possible)					
	1	Not applicable, there is no organised continuing education			0.000	0.00	0.00
	2	General practitioners			0.250	0.00	0.25
	3	Practice nurses	x		0.250	0.25	0.25
	4	Physician assistants	x		0.250	0.25	0.25
	5	Endocrinologists	x		0.250	0.25	0.25
	6	Diabetes nurses	x		0.250	0.25	0.25
	7	Dieticians	x		0.250	0.25	0.25
	8	Ophthalmologists	x		0.250	0.25	0.25
	9	Optometrists	x		0.250	0.25	0.25
	10	Podiatrists	x		0.250	0.25	0.25
	11	Other, namely		namely…………..	0.250	0.00	0.25
				With 4 care providers, the maximum score is reached		**1.00**	**1.00**
**5.8**	Is there a recorded protocol on how incidents should be reported?						
		(choose the best possible answer)					
	1	No, this is the responsibility of the individual care provider			0.000	0.00	0.00

	2	No, this is under development					0.333	0.00	0.33
	3	Yes, incidents are reported to the care provider who is in charge					0.667	0.00	0.67
	4	Yes, incidents are reported to the care provider who is in charge as well as to the care group	x				1.000	1.00	1.00
								**1.00**	**1.00**

**5.9**	Is the care groups using a system that systematically scans and alerts the care provider when a patient may be experiencing care-related harm?								
		(choose the best possible answer)							
	1	No, this is not used					0.000	0.00	0.00
	2	This is occasionally used by some care providers in the care group					0.333	0.00	0.33
	3	No, this is in development for all care providers in the care group					0.667	0.00	0.67
	4	Yes, this is operational throughout the care group	x				1.000	1.00	1.00
								**1.00**	**1.00**

**5.10**	Is there a policy aiming at better health care for distinct subgroups within your care group (e.g. for people with kidney problems)?								
		(choose the best possible answer)							
	1	No, this distinction is not made in the care group					0.000	0.00	0.00
	2	This is occasionally being done in the care group					0.333	0.00	0.33
	3	In the care group is structurally aiming at distinct subgroups					0.667	0.00	0.67
	4	The care group is structurally aiming at distinct subgroups and has adopted a policy for this	x				1.000	1.00	1.00
								**1.00**	**1.00**

**5.11**	Does your care group have a special policy to provide proper care for patient groups who are hard to reach and / or for complex patient groups?								
		(several answers possible)							
	1	No, there is no such policy					0.000	0.00	0.00
	2	Yes, there is a special policy for people with low socioeconomic status	x				0.167	0.17	0.17
	3	Yes, for people from ethnic minorities					0.167	0.00	0.17
	4	Yes, for people who structurally avoid care	x				0.167	0.17	0.17
	5	Yes, for the less mobile					0.167	0.00	0.17
	6	Yes, for people who use multiple drugs (polypharmacy)	x				0.167	0.17	0.17
	7	Yes, for people with multimorbidity					0.167	0.00	0.17
	8	Other, namely		namely……………			0.000	0.00	0.00
								**0.50**	**1.00**

	Question number	score achieved	maximum points	achieved score	experts' weighting	achieved weighted score	weighted maximum score
Elements of quality improvement	5.1	0.60	1	60%	10%	6%	9%
Feedback/benchmark	5.2 and 5.3	2.00	2	100%	25%	25%	18%
Visitation	5.4 and 5.5	1.80	2	90%	25%	23%	18%
Education	5.6 and 5.7	2.00	2	100%	20%	20%	18%
Patient safety	5.8 and 5.9	2.00	2	100%	10%	10%	18%
Sub groups	5.10 and 5.11	1.50	2	75%	10%	8%	18%
**TOTAL SCORE Quality improvement policy**		**9.90**	**11**	90%	100%	91%	100%

6. Management strategies

**6.1**	Who is the leader of quality management in your organisation?								
		(choose the best possible answer)							
	1	No one					0.000	0.00	0.00
	2	A general practitioner with specialty in diabetes care					1.000	0.00	1.00
	3	A steering committee or commission					1.000	0.00	1.00
	4	A quality officer	x				1.000	1.00	1.00
	5	An outside company or consultant					1.000	0.00	1.00
	6	Representatives of care providers					1.000	0.00	1.00
	7	The management					1.000	0.00	1.00
	8	Other, namely		namely……………			1.000	0.00	1.00
								**1.00**	**1.00**

**6.2**	How is the quality policy <u>structurally</u> embedded in your organisation?								
		(several answers possible)							
	1	It is not structurally embedded					0.000	0.00	0.00

	2	There is a special internal budget set aside for quality policy	x				0.250	0.25	0.25
	3	One or more quality officers are appointed					0.250	0.00	0.25
	4	There is an outside company or consultant involved	x				0.250	0.25	0.25
	5	There is an expert team / a quality team of general practitioners with specialty in diabetes care, diabetes consultants and / or endocrinologists					0.250	0.00	0.25
	6	Other, namely		namely……………			0.000	0.00	0.00
								**0.50**	**1.00**
**6.3**	Does your care group use any form of cyclic quality policy as a tool to improve the quality of diabetes care?								
		(choose the best possible answer)							
	1	No					0.000	0.00	0.00
	2	Yes, but occasionally					0.333	0.00	0.33
	3	Yes, now occasionally, but structural use under development	x				0.667	0.67	0.67
	4	Yes, we structurally use cyclic quality policy					1.000	0.00	1.00
								**0.67**	**1.00**
**6.4**	Does your care group use a certified quality system?								
		(choose the best possible answer)							
	1	Yes, namely:	x				1.000	1.00	1.00
	2	ISO					0.000	0.00	0.00
	3	INK	x				0.000	0.00	0.00
	4	HKZ					0.000	0.00	0.00
	5	Other, namely		……………………			0.000	0.00	0.00
	6	No, only on general practice level, a quality system is used					0.667	0.00	0.67
	6	No, the providers have to meet specific qualifications for their care profession					0.333	0.00	0.33
	7	No					0.000	0.00	0.00
								**1.00**	**1.00**
**6.5**	Please indicate which of the following documents are available in your care group?								
			**No**	**Under development**	**Yes**				
		(choose the best possible answer)	0.000	0.100	0.200				
	1	**Mission document**: vision and priorities of the care group	x					0.00	0.20
	2	**Quality action plan** for the care group: measures for the implementation and planning of actions to achieve quality goals		x				0.10	0.20
	3	**Annual quality report**: reporting on all activities carried out to ensure quality of care and its outcomes			x			0.20	0.20
	4	**Quality manual**: description of all procedures used by care group and the individuals responsible to ensure quality of care		x				0.10	0.20
	5	Is the quality manual available to all employees within the care group?			x			0.20	0.20
								**0.60**	**1.00**
			Question number	score achieved	maximum points	achieved score	experts' weighting	achieved weighted score	weighted maximum score
		Structural policy	6.1-6.3	2.17	3	72%	40%	29%	60%
		Quality system	6.4	1.00	1	100%	40%	40%	20%
		Quality documents	6.5	0.60	1	60%	20%	12%	20%
		**TOTAL SCORE Management strategies**		**3.77**	**5**	75%	100%	81%	100%
