# Supplementary material for: Diabetes quality management in Dutch care groups and outpatient clinics: a cross-sectional study
Source: BMC Res Notes. 2014 Aug 7;7:497. doi: 10.1186/1756-0500-7-497 (PMC4132241; doi:10.1186/1756-0500-7-497)
Supplement: Additional file 4 — Scoring of questionnaire for diabetes outpatient clinics. [file 1756-0500-7-497-S4.pdf]

Outpatient clinics

Legend
- orange: Weighting factor
- red: special scoring (maximised)
- **Achieved score of this outpatient clinic**
- **Maximum possible score**

1. Organisation of care

							score	max score
**1.1**	The diabetes care program							
		No	Yes	Under development				
	(choose the best possible answer)	0.000	0.167	0.083				
1	Is with respect to the protocol recorded in writing		x				0.17	0.17
2	Its contents is based on evidence-based standards and guidelines		x				0.17	0.17
3	Is with regard to the agreements on organisation and implementation recorded in writing		x				0.17	0.17
4	Includes a description of the functions of all the different care providers		x				0.17	0.17
5	Is available in writing for all care providers involved		x		*)		0.17	0.17
6	Is structurally maintained and updated	x			*)			
7	Contains a description of all the steps followed by the patient follows (care pathway)		x				0.17	0.17
	*) The maximum score of sub question 5 and 6 counts						**1.00**	**1.00**

							score	max score
**1.2**	Who are involved in the designing, implementing and/or evaluation of the diabetes care program?							
		designing	implementing	evaluating and adapting	Not involved			
	(Please tick all that apply, more answers possible)	0.056	0.111	0.167	0.000			
1	The medical staff				x		0.00	0.17
2	An endocrinologist or internist with the most affinity with diabetes	x	x	x			0.17	0.17
3	Diabetes nurses	x	x	x			0.17	0.17
4	A diabetes commission	x	x	x			0.17	0.17
5	A quality officer	x		x			0.17	0.17
6	Representative(s) of patients				x		0.00	0.17
7	Other, namely …..				x		0.00	0.17
	horizontal maximum score counts						**1.00**	**1.00**

					score	max score
**1.3**	How often is the care program being evaluated?					
	(choose the best possible answer)					
1	Not applicable. The care program is not being evaluated			0.000	0.00	0.00
2	The care program is not being evaluated with a regular frequency			0.500	0.00	0.50
3	Annually	x		1.000	1.00	1.00
4	Biannually			1.000	0.00	1.00
5	Otherwise, namely		namely	0.500	0.00	0.50
	total score maximised on 1 point				**1.00**	**1.00**

					score	max score
**1.4**	The content of the care program is adjusted if:					
	(several answers possible)					
1	Not applicable. The content of the care program is being not adjusted	x		0.000	0.00	0.00
2	The standard of the Dutch endocrinologists (NIV) is adjusted		*)	0.500	0.00	0.50
3	The care standard of the Dutch Diabetes Federation (NDF) is revised		*)	0.500	0.00	0.50
4	New evidence-based guidelines are available	x	*)	0.500	0.50	0.50
5	There are new medications coming on the market		**)	0.500	0.00	0.50
6	The content of the contract regarding the multi-agency healthcare (keten-dbc) has changed		**)	0.500	0.00	0.50
7	Outcomes / results of care give cause	x	**)	0.500	0.50	0.50
8	Otherwise, namely		namely	0.500	0.00	0.50
	*) The sum of sub questions 2, 3 and 4 is maximised on 0,5 point				**1.00**	**1.00**
	**) The sum of sub questions 5, 6 and 7 is maximised on 0,5 point					

				score	max score
**1.5**	Does the diabetes outpatient clinic have a policy on coordination of care?				
	(choose the best possible answer)				
1	Coordination of care is not a priority within the diabetes outpatient clinic		0.000	0.00	0.00
2	Coordination of care is under development within the diabetes outpatient clinic	x	0.500	0.50	0.50
3	The diabetes outpatient clinic has one care coordinator per patient, namely:		0.000	0.00	0.00
3-a	The endocrinologist is always the care coordinator		1.000	0.00	1.00
3-b	A diabetes nurse in consultation with the endocrinologist, acts as care coordinator		1.000	0.00	1.00
4	Otherwise, namely		1.000	0.00	1.00
				**0.50**	**1.00**

1.6 To what extent do the following statements regarding continuity of care apply to your diabetes outpatient clinic?

			Not at all				Yes, completely			
			1	2	3	4	5			
		(choose the best possible answer)	0.000	0.031	0.063	0.094	0.125			
	1	In the diabetes clinic, patients received univocal advice from the different care providers about diabetes?					x		0.13	0.13
	2	Referral from primary care to the diabetes outpatient clinic is consistent with regional multi-agency health care agreements.					x		0.13	0.13
	3	The referral from the diabetes outpatient clinic to primary care is consistent with regional multi-agency health care agreements.				x			0.09	0.13
	4	The diabetes outpatient clinic promotes the structural planning of care appointments for patients.				x			0.09	0.13
	5	The diabetes outpatient clinic promotes the use of a call system for calling patients.		x					0.03	0.13
	6	The diabetes outpatient clinic has insight into patients who stay away from their care provider (turnover of the patients).				x			0.09	0.13
	7	The diabetes outpatient clinic encourages the use of a call system for no show contacts.				x			0.09	0.13
	8	The diabetes outpatient clinic has insight into the number of patients that quit treatment by a care provider (by death, moving or transferral to primary care).				x			0.09	0.13
									**0.75**	**1.00**

1.7 Information and communication system

			Yes	Under development	no					
		(choose the best possible answer)	0.100	0.050	0.000					
	1	Within the diabetes outpatient clinic is one method of recording (for example, standardisation of care outcomes)	x						0.10	0.10
	2	There is one joint health record for a patient for all care providers within the diabetes outpatient clinic.	x						0.10	0.10
	3	The patient file is also accessible to care providers in the hospital outside the diabetes outpatient clinic			x				0.00	0.10
	4	There are one or more shared information systems, such as Chain Information Systems (KIS) with care providers outside the diabetes outpatient clinic			x				0.00	0.10
	5	Does the diabetes outpatient clinic have access to information of general practitioners			x				0.00	0.10
	6	An individual care plan can be registered in the patient record	x						0.10	0.10
	7	Individual self-management targets can be registered in the patient record			x				0.00	0.10
	8	Are care providers being reminded to follow protocol by the hospital information system (ZIS)			x				0.00	0.10
	9	There is one way of reporting for all care provided within the diabetes outpatient clinic	x						0.10	0.10
	10	There is unity in referral forms within the diabetes outpatient clinic	x						0.10	0.10
									**0.50**	**1.00**

1.8 Is your diabetes outpatient clinic using electronic health records?

		(choose the best possible answer)								
	1	Yes, diabetes outpatient clinic exclusively works with an electronic patient record	x					1.000	1.00	1.00
	2	The diabetes outpatient operates with a paper patient record supplemented with an electronic file with for example file containing lab results and correspondence.						0.000	0.00	0.00
									**1.00**	**1.00**

1.9 Which care providers do have access to the medical record?

			Only a paper record	Only electronic record	Both	No access				
		(choose the best possible answer)	0.100	0.200	0.200	0.000				
	1	Endocrinologist			x				0.20	0.20
	2	Diabetes nurse			x				0.20	0.20
	3	Cardiologist				x			0.00	0.20
	4	Nephrologist				x			0.00	0.20
	5	Ophthalmologist				x			0.00	0.20
	6	Optometrist				x			0.00	0.20
	7	Pharmacist				x			0.00	0.20
	8	Dietician			x				0.20	0.20
	9	Podiatrist				x			0.00	0.20
	10	Psychologist				x			0.00	0.20
	11	Physiotherapist				x			0.00	0.20
	12	General Practitioner and Practice nurse				x			0.00	0.20
	13	……………………							0.00	0.20
					5 care providers gives maximum of 1 point				**0.60**	**1.00**

	Question number	score achieved	maximum points	achieved score	experts' weighting	achieved weighted score	weighted maximum score
1. Care program	1.1-1.4	4.00	4	100%	35%	35%	44%
2. Continuity and coordination	1.5 and 1.6	1.25	2	63%	35%	22%	22%
3. Communication and information	1.7-1.9	2.10	3	70%	30%	21%	33%
**TOTAL SCORE Organisation of care**		**7.35**	**9**	**82%**	100%	78%	100%

2. Multidisciplinary teamwork

2.1 Which care providers / organisation have written work agreements concerning the diabetes care program with your diabetes outpatient clinic?

	(several answers apply)			experts' weighting	achieved weighted score	weighted maximum score
1	Endocrinologist	x		0.059	0.06	0.06
2	Diabetes Nurse	x		0.059	0.06	0.06
3	General practitioner and practice nurse	x		0.059	0.06	0.06
4	Dietician	x		0.059	0.06	0.06
5	Ophthalmologist	x		0.059	0.06	0.06
6	Optometrist			0.059	0.00	0.06
7	Nephrologist			0.059	0.00	0.06
8	Cardiologist			0.059	0.00	0.06
9	Vascular surgeon			0.059	0.00	0.06
10	Rehabilitation physician			0.059	0.00	0.06
11	Podiatrist			0.059	0.00	0.06
12	Psychologist	x		0.059	0.06	0.06
13	Pharmacist			0.059	0.00	0.06
14	Physiotherapist			0.059	0.00	0.06
15	Residential homes			0.059	0.00	0.06
16	Nursing homes			0.059	0.00	0.06
17	Other, namely....		namely	0.059	0.00	0.06
					**0.35**	**1.00**

2.2 How does the outpatient clinic facilitate multidisciplinary teamwork?

		Yes	Under development	No	achieved weighted score	weighted maximum score
	(choose the best possible answer)	0.167	0.083	0.000		
1	The diabetes outpatient clinic has protocols on which care should be provided by which care provider	x			0.17	0.17
2	There are agreements about the medical responsibility for task substitution (e.g. a diabetes nurse taking over tasks of an endocrinologist)	x			0.17	0.17
3	The outpatient clinic promotes multidisciplinary consultation on diabetes patients (structural consultation between at least two collaborating care providers with different expertise)	x			0.17	0.17
4	The diabetes outpatient clinic has a shared vision on education (goal and strategy)			x	0.00	0.17
5	The diabetes outpatient clinics periodically organises joint training for care providers involved		x		0.08	0.17
6	Care providers from different disciplines are providing joint consultation		x		0.08	0.17
					**0.67**	**1.00**

2.3 What kind of cooperation is there in your outpatient clinic? Has this been defined in a protocol? Is this cooperation being evaluated and are the results of this evaluation being used to improve the quality of the cooperation?

		Do not exist/does not take place	Is not yet defined in protocol	Are defined in protocol	Are periodically evaluated	Are used in improvement efforts	achieved weighted score	weighted maximum score
	(choose the best possible answer)	0.000	0.025	0.050	0.075	0.111		
1	The diabetes outpatient clinic has agreements on cooperation between care providers within the hospital			x			0.05	0.11
2	The diabetes outpatient clinic has agreements on transfer of patient between care providers within the hospital				x		0.08	0.11
3	The diabetes outpatient clinic has agreements on transfer of patient between care providers outside the hospital					x	0.11	0.11
4	The outpatient has agreed on multidisciplinary referral and back referral criteria (e.g. to dietician and GP)					x	0.11	0.11
5	There is structurally multidisciplinary consultation on the treatment of diabetes patients in the diabetes outpatient clinic				x		0.08	0.11
6	The diabetes outpatient clinic has agreements with other medical specialist concerning multimorbidity				x		0.08	0.11
7	The outpatient clinic organises regular meetings to discuss guidelines / standards		x				0.03	0.11
8	The outpatient clinic organises regular meetings to discuss roles and responsibilities				x		0.08	0.11

No.	Item						Weighting	Score	Max
9	The diabetes outpatient clinic organises regular meetings to discuss problems concerning diabetes care.					x		0.11	0.11
10	Otherwise, namely ………………						.	0.00	0.11
								**0.71**	**1.00**

2.4 Does the diabetes outpatient clinic have a multidisciplinary foot team?

No.	(several answers apply)	Answer		Weighting	Score	Max
1	No, there is no multidisciplinary foot team			0.000	0.00	0.00
2	A multidisciplinary foot team under development			0.333	0.00	0.33
3	Yes, there is a multidisciplinary foot team, in which the following care providers participate:	x		0.000	0.00	0.00
3-a	Endocrinologist	x		0.111	0.11	0.11
3-b	(Vascular) surgeon	x		0.111	0.11	0.11
3-c	Diabetes nurse	x		0.111	0.11	0.11
3-d	Podiatrist	x		0.111	0.11	0.11
3-e	Plaster cast expert	x		0.111	0.11	0.11
3-f	Rehabilitation specialist	x		0.111	0.11	0.11
3-g	Orthopaedic surgeon			0.111	0.00	0.11
3-h	Orthopaedic shoemaker			0.111	0.00	0.11
3-i	Other, namely		namely	0.111	0.00	0.11
					**0.67**	**1.00**

	Question number	score achieved	maximum points	achieved score	experts' weighting	achieved weighted score	weighted maximum score
Work agreement	2.1; 2.4;	0.43	1.11	39%	10%	4%	28%
Tasks and responsibilities	2.2.1; 2.2.2; 2.3.7	0.43	0.56	78%	20%	16%	14%
Teamwork/consultation/shared education/guidelines	2.2.3-6; 2.3.1; 2.3.5; 2.3.9; 2.3.10;	0.57	1.11	51%	30%	15%	111%
Transfer and referral	2.3.2; 2.3.3; 2.3.4	0.30	0.33	89%	20%	18%	8%
Foot team	2.4	0.67	1.00	67%	20%	13%	25%
**TOTAL SCORE Multidisciplinary teamwork**		**2.39**	**4.00**	60%	100%	66%	100%

3. Patient centeredness

3.1 How is self-management in diabetic patients supported by the outpatient clinic:

No.	(Please tick all that apply)	Answer		Weighting	Score	Max
1	Is not supported by the outpatient clinic			0.000	0.00	0.00
2	Support is currently under development	x		0.250	0.25	0.25
3	Dissemination of information (brochures, booklets)	x		0.500	0.50	0.50
4	Courses for care providers			0.750	0.00	0.75
5	Courses for care patients			1.000	0.00	1.00
6	Otherwise, namely		namely: ……………………………..	0.250	0.00	0.25
				Maximum score counts	**0.50**	**1.00**

3.2 The use of individual care plans

No.	(choose the best possible answer)	Answer	Weighting	Score	Max
1	Is not stimulated by the outpatient clinic		0.000	0.00	0.00
2	Is under development within the outpatient clinic	x	0.333	0.33	0.33
3	Is actively encouraged by the outpatient clinic		0.667	0.00	0.67
4	Is actively encouraged within the outpatient clinic and periodically evaluated on the basis of predetermined goals		1.000	0.00	1.00
				**0.33**	**1.00**

3.3 The policy of the outpatient clinic regarding patient education is:

No.	(choose the best possible answer)	Answer	Weighting	Score	Max
1	Not developed within the outpatient clinic		0.000	0.00	0.00
2	In development within the outpatient clinic		0.250	0.00	0.25
3	To achieve as uniform information as possible by means of coordination		0.500	0.00	0.50
4	To achieve as uniform information as possible by means of coordination and also to train care givers in uniform information	x	1.000	1.00	1.00
				**1.00**	**1.00**

3.4 How does the patient have access to her/his medical records?

No.	(choose the best possible answer)	Answer	Weighting	Score	Max
1	The patient only has access to medical records if he / she expressly requests it.	x	0.000	0.00	0.00
2	Access to medical data is under development		0.333	0.00	0.33
3	The patient can see the data in a diabetes passport		0.667	0.00	0.67
4	Patients can view their information through a patient portal		1.000	0.00	1.00
				**0.00**	**1.00**

**3.5**	Can the patient data add to her/his electronic records?								
	(choose the best possible answer)								
1	No, there is no electronic record						0.000	0.00	0.00
2	There is an electronic file, but patient can not add data						0.333	0.00	0.33
3	The ability of the patient to add data itself is under development	x					0.667	0.67	0.67
4	Yes, through a patient portal						1.000	0.00	1.00
								**0.67**	**1.00**

		Yes	Under development	No	I do not know		
**3.6**	Patient interests						
	(choose the best possible answer)	0.167	0.083	0.000	0.000		
1	Is there an established protocol that the patient is informed of guidelines / standards (e.g. "de diabetes zorgwijzer)				x	0.00	0.17
2	Is there a central location (front-office) where the patient can ask questions? (a desk, a central phone number or a website)	x				0.17	0.17
3	Are consultation hours are from different care providers coordinated and tuned?		x			0.08	0.17
4	Is there a fixed designated person to whom the patient can address his/her questions?		x			0.08	0.17
5	Is the patient privacy guaranteed in a multidisciplinary care record?	x				0.17	0.17
6	Is the privacy of the patient guaranteed in the collection of data, for example for a benchmark?		x			0.08	0.17
						**0.58**	**1.00**

**3.7**	How are patients involved in your diabetes outpatient clinic? By means of					
	(several answers apply)					
1	Client-board			0.200	0.00	0.20
2	A complaints committee			0.200	0.00	0.20
3	Structural cooperation with regional patient / consumer federation			0.200	0.00	0.20
4	Structural cooperation with the national patient organisation (Dutch diabetes cooperation)	x		0.200	0.20	0.20
5	Patients are not structurally involved in the outpatient clinic, but we are preparing to do so.			0.100	0.00	0.10
6	Patients are not involved in the diabetes outpatient clinic			0.000	0.00	0.00
7	Otherwise, namely		namely: ……………………………...	0.100	0.00	0.10
					**0.20**	**1.00**

	Question number	score achieved	maximum points	achieved score	experts' weighting	achieved weighted score	weighted maximum score
Self-management	3.1	0.50	1	50%	20%	10%	14%
Individual care plan	3.2	0.33	1	33%	20%	7%	14%
Policy on patient education	3.3	1.00	1	100%	20%	20%	14%
Inspection of medical file	3.4 and 3.5	0.67	2	33%	12%	4%	29%
Patient interests	3.6	0.58	1	58%	18%	11%	14%
Patient involvement	3.7	0.20	1	20%	10%	2%	14%
**TOTAL SCORE Patient centeredness**		**3.28**	**7**	47%	100%	53%	100%

4. Performance management

**4.1**	Are registered data of different care providers collected for feedback or a benchmark?				
	(choose the best possible answer)				
1	Yes, continue with question 4.2	x	0.000	0.00	0.00
2	No, continue with question 5.1		0.000	0.00	0.00
				**0.00**	**0.00**

		By an electronic information system	By a paper file	Are not collected			
**4.2**	How are the records of the following care providers for feedback or benchmark collected for the diabetes?						
	(choose the best possible answer)	0.091	0.045	0.000			
1	Endocrinologist	x				0.09	0.09
2	Diabetes nurse	x				0.09	0.09
3	Dietician	x				0.09	0.09
4	Ophthalmologist					0.00	0.09
5	Optometrist					0.00	0.09
6	Nephrologist					0.00	0.09
7	Cardiologist					0.00	0.09
8	Podiatrist					0.00	0.09
9	Psychologist					0.00	0.09
10	Physiotherapist					0.00	0.09
11	Other, namely				namely: ……………	0.00	0.09
						**0.27**	**1.00**

4.3 How has the diabetes outpatient clinic organised the checking of correctness of the data recorded and supplied by care providers?

	(several answers apply)					
1	The outpatient clinic has not organised anything for this			0.000	0.00	0.00
2	This is done by the caregivers themselves	x		0.333	0.33	0.33
3	The outpatient clinic outsources this to an independent organisation			0.500	0.00	0.50
4	In the information system has integrated alerts to prevent erroneous data			0.500	0.00	0.50
5	Otherwise, namely		namely: …………..	0.333	0.00	0.33
			Total score maximised on 1		0.33	1.00

4.4 Who edits the submitted results data from the providers to feedback data / **internal** indicators?

	(choose the best possible answer)					
1	The respective care providers do this themselves	x		0.333	0.33	0.33
2	The outpatient clinic does this			0.667	0.00	0.67
3	The outpatient clinic outsources this out to an independent organisation			1.000	0.00	1.00
4	Otherwise, namely		namely: …………..	0.333	0.00	0.33
					0.33	1.00

4.5 Who processes the data supplied from the providers to **external** accountability indicators, for example Visible Care (ZIZO) or insurers?

	(choose the best possible answer)					
1	The respective providers do this themselves	x		0.333	0.33	0.33
2	The outpatient clinic does this			0.667	0.00	0.67
3	The outpatient clinic outsources this out to an independent organisation			1.000	0.00	1.00
4	Otherwise, namely		namely:….	0.333	0.00	0.33
					0.33	1.00

4.6 At what level are the data analysed?

	(several answers apply)					
1	Data are not analysed			0.000	0.00	0.00
2	At the patient level	x		0.333	0.33	0.33
3	At care provider level			0.333	0.00	0.33
4	At the level of the outpatient clinic	x		0.333	0.33	0.33
					0.67	1.00

4.7 How are the data analysed

	(several answers apply)					
1	Data are not analysed			0.000	0.00	0.00
2	Only averages are determined	x		0.330	0.33	0.33
3	Both averages and dispersion are determined			0.500	0.00	0.50
4	Also individual extreme values are determined			0.660	0.00	0.66
5	On the basis of the use of medication sub-groups will be determined, and data are analysed at that level			0.500	0.00	0.50
6	Based on demographic data subsets are determined and data are analysed at that level			0.500	0.00	0.50
7	Other, namely		……………………..	0.167	0.00	0.17
			Total score maximised on 1 point		0.33	1.00

4.8 Which dataset is recorded in the outpatient clinic?

	(several answers apply)					
1	There is no specific data set recorded			0.000	0.00	0.00
2	Basic set of performance indicators Hospital 2005			0.500	0.00	0.50
3	The minimum data set (MDS) of the Dutch Healthcare Authority (Nza), Visible Care	x		0.500	0.50	0.50
4	The e-Diabetes core set of the Dutch Diabetes Federation (NDF)			0.500	0.00	0.50
5	Indicators of the Dutch General Practitioners (NHG)			0.500	0.00	0.50
6	Otherwise, namely		……………………..	0.500	0.00	0.50
			Total score maximised on 1 point		0.50	1.00

4.9 Which indicators are calculated?

	(several answers apply)					
1	Visible care (ZIZO) indicators	x		0.500	0.50	0.50
2	"Quality of care in the window" indicators; indicators of the Dutch internist association (NIV)			0.500	0.00	0.50
3	Indicators for insurers			0.500	0.00	0.50
4	Indicators of the Dutch General Practitioners (NHG)			0.500	0.00	0.50
5	Other, namely		……………………..	0.500	0.00	0.50
			Total score maximised on 1 point		0.50	1.00

	Question number	score achieved	maximum points	achieved score	experts' weighting	achieved weighted score	weighted maximum score
Registering results	4.1 and 4.2	0.27	1	27%	30%	8%	13%

Control of results	4.3	0.33	1	33%		10%	3%	13%
Processing of results	4.4 and 4.5	0.67	2	33%		10%	3%	25%
Analysing results	4.6 and 4.7	1.00	2	50%		20%	10%	25%
Measured outcomes	4.8 and 4.9	1.00	2	50%		30%	15%	25%
**TOTAL SCORE Performance management**		**3.27**	**8**	41%		100%	40%	100%

5. Quality improvement policy

5.1 What results does your organisation use for quality improvements?

		Not measured	Measured! Used for quality: Yes	Measured! Used for quality: No		
	(choose the best possible answer)	0.000	0.200	0.100		
1	Performance indicators		x		0.20	0.20
2	Experiences of the patient: for example, by asking patients to complete the CQ index		x		0.20	0.20
3	Views of the referrers or other partners in sequenced care: e.g. satisfaction surveys or evaluation		x		0.20	0.20
4	Complaints registration: the diabetes outpatient clinic has an overview of all complaints about diabetes care that are received the outpatient clinic		x		0.20	0.20
5	Waiting times for care providers: are there waiting lists / waiting times for access to care providers within the diabetes outpatient clinic		x		0.20	0.20
5-a	If there are any waiting times: :					
	for which care provider(s) / how long?:	………………………………………………/…………………				
		………………………………………………/…………………				
		………………………………………………/…………………				
					**1.00**	**1.00**

5.2 With which care providers is the feedback information discussed?

	(several answers apply)				
1	Not applicable. There is no feedback information offered.		0.000	0.00	0.00
2	Endocrinologists	x	0.200	0.20	0.20
3	Diabetes nurses	x	0.200	0.20	0.20
4	Dieticians		0.200	0.00	0.20
5	Podiatrists		0.200	0.00	0.20
6	Ophthalmologists		0.200	0.00	0.20
7	Optometrist		0.200	0.00	0.20
8	Cardiologists		0.200	0.00	0.20
9	Nephrologists		0.200	0.00	0.20
10	(Vascular)surgeons		0.200	0.00	0.20
11	Psychologists		0.200	0.00	0.20
12	Physiotherapists		0.200	0.00	0.20
13	Pharmacists		0.200	0.00	0.20
14	Other, namely	……………………	0.200	0.00	0.20
			Total score is maximised on 1 point	**0.40**	**1.00**

5.3 Is benchmarking being used in your care group to make improvements?

	(choose the best possible answer)				
1	No, benchmarking is not being used		0.000	0.00	0.00
2	Benchmarking is occasionally used	x	0.333	0.33	0.33
3	The policy to structurally use benchmarking for quality improvement is still under development		0.667	0.00	0.67
4	Benchmarking is structurally used for quality improvement		1.000	0.00	1.00
				**0.33**	**1.00**

5.4 Which care providers are periodically inspected?

	(several answers apply)				
1	There is no or only occasional inspection		0.000	0.00	0.00
2	Endocrinologists	x	0.125	0.13	0.13
3	Diabetes nurses		0.125	0.00	0.13
4	Dieticians		0.125	0.00	0.13
5	Ophthalmologists	x	0.125	0.13	0.13
6	Optometrist		0.125	0.00	0.13
7	Cardiologists	x	0.125	0.13	0.13
8	Nephrologists	x	0.125	0.13	0.13
9	Other, namely	……………………	0.125	0.00	0.13
				**0.50**	**1.00**

5.5 How is the training policy designed?

	(choose the best possible answer)				
1	Education is not regarded as a responsibility of the organisation but of the individual care provider	x	0.000	0.00	0.00
2	The training policy within the diabetes outpatient clinic is under development		0.333	0.00	0.33
3	The diabetes outpatient clinic has a training policy for all care providers		0.667	0.00	0.67

	4	The diabetes outpatient clinic has a training policy for all care providers. This is regularly updated.			1.000	0.00	1.00
						**0.00**	**1.00**
**5.6**	For which care providers has continuing education been organised in the past year ?						
		(several answers apply)					
	1	Not applicable, there is no organised continuing education			0.000	0.00	0.00
	2	Endocrinologists	x		0.250	0.25	0.25
	3	Diabetes nurses	x		0.250	0.25	0.25
	4	GPs	x		0.250	0.25	0.25
	5	Practice nurses in a general practice	x		0.250	0.25	0.25
	6	Dieticians			0.250	0.00	0.25
	7	Ophthalmologists			0.250	0.00	0.25
	8	Optometrists			0.250	0.00	0.25
	9	Podiatrists			0.250	0.00	0.25
	10	Cardiologists			0.250	0.00	0.25
	11	Psychologists			0.250	0.00	0.25
	12	Other, namely		……………………	0.250	0.00	0.25
				Total score maximised on 1 point		**1.00**	**1.00**
**5.7**	Is there a recorded protocol on how incidents should be reported?						
		(choose the best possible answer)					
	1	No, this is the responsibility of the individual care provider			0.000	0.00	0.00
	2	No, this is under development			0.333	0.00	0.33
	3	Yes, incidents are reported to the care provider who is in charge			0.667	0.00	0.67
	4	Yes, incidents are reported to the care provider who is in charge as well as to the diabetes outpatient clinic	x		1.000	1.00	1.00
						**1.00**	**1.00**
**5.8**	Is the outpatient clinic using a system that systematically scans and alerts the care provider when a patient may be experiencing care-related harm?						
		(choose the best possible answer)					
	1	No, this is not used			0.000	0.00	0.00
	2	This is occasionally used by some care providers in the outpatient clinic			0.333	0.00	0.33
	3	No, this is under development for all care providers in the outpatient clinic			0.667	0.00	0.67
	4	Yes, this is operational for all care providers in the diabetes outpatient clinic	x		1.000	1.00	1.00
						**1.00**	**1.00**
**5.9**	How is the monitoring of the medication record organised?						
		(choose the best possible answer)					
	1	This is not regulated			0.000	0.00	0.00
	2	Through the file of the GP			1.000	0.00	1.00
	3	Through the medical passport of the patient			1.000	0.00	1.00
	4	Through the pharmacy of the patient			1.000	0.00	1.00
	5	Through the hospital pharmacy	x		1.000	1.00	1.00
						**1.00**	**1.00**
**5.10**	Is there a policy aiming at better health care for distinct subgroups within your diabetes outpatient clinic (e.g. for people with kidney problems)?						
		(choose the best possible answer)					
	1	No, this distinction is not made			0.000	0.00	0.00
	2	This distinction is occasionally made			0.333	0.00	0.33
	3	The diabetes clinic structurally distinguishes subgroups of patients			0.667	0.00	0.67
	4	The outpatient clinic is structurally aiming at distinct subgroups and if possible there is developed a policy for this	x		1.000	1.00	1.00
						**1.00**	**1.00**
**5.11**	Does your outpatient clinic have a special policy to provide proper care for patient groups who are hard to reach?						
		(several answers apply)					
	1	No, there is no such policy			0.000	0.00	0.00
	2	Yes, there is a special policy for people with low socioeconomic status	x		0.143	0.14	0.14
	3	Yes, for people from ethnic minorities			0.143	0.00	0.14
	4	Yes, for people who structurally avoid care	x		0.143	0.14	0.14
	5	Yes, for the less mobile			0.143	0.00	0.14
	6	Yes, for people who use multiple drugs (polypharmacy)			0.143	0.00	0.14
	7	Yes, for people with multimorbidity			0.143	0.00	0.14
	8	Yes, for people who visit multiple specialists			0.143	0.00	0.14
	9	Other, namely		……………………	0.000	0.00	0.00
						**0.29**	**1.00**

	Question number	score achieved	maximum points	achieved score	experts' weighting	achieved weighted score	weighted maximum score
Elements of quality improvement	5.1	1.00	1	100%	10%	10%	9%
Feedback/benchmark	5.2 and 5.3	0.73	2	37%	25%	9%	18%
Visitation	5.4	0.50	1	50%	25%	13%	9%
Education	5.5 and 5.6	1.00	2	50%	20%	10%	18%
Patient safety	5.7 - 5.9	3.00	3	100%	10%	10%	27%
Sub groups	5.10 and	1.29	2	64%	10%	6%	18%
**TOTAL SCORE Quality improvement policy**		7.52	11	68%	100%	58%	100%

6. Management strategies

6.1 Who is the leader of quality management in your organisation?

	(choose the best possible answer)			experts' weighting	achieved weighted score	weighted maximum score
1	No one			0.000	0.00	0.00
2	A steering committee or commission			1.000	0.00	1.00
3	A quality officer			1.000	0.00	1.00
4	An outside company or consultant			1.000	0.00	1.00
5	Medical staff			1.000	0.00	1.00
6	The management of the hospital			1.000	0.00	1.00
7	The head of the outpatient clinic			1.000	0.00	1.00
8	All internists together			1.000	0.00	1.00
9	The internist that is the most specialised in diabetes (endocrinologist)	x		1.000	1.00	1.00
10	All internists that are specialised in diabetes care together			1.000	0.00	1.00
11	Other, namely		……………………	1.000	0.00	1.00
					1.00	1.00

6.2 How is the quality policy structurally embedded in your organisation?

	(several answers apply)			experts' weighting	achieved weighted score	weighted maximum score
1	It is not structurally embedded			0.000	0.00	0.00
2	There is a special internal budget set aside for quality policy			0.250	0.00	0.25
3	One or more steering committees (of commissions) have been appointed	x		0.250	0.25	0.25
4	One or more quality officers are appointed	x		0.250	0.25	0.25
5	There is an outside company or consultant involved			0.250	0.00	0.25
6	Other, namely		……………………	0.000	0.00	0.00
					0.50	1.00

6.3 Does your diabetes outpatient clinic use any form of cyclic quality policy as a tool to improve the quality of

	(choose the best possible answer)		experts' weighting	achieved weighted score	weighted maximum score
1	No		0.000	0.00	0.00
2	Yes, but occasionally		0.333	0.00	0.33
3	Yes, now occasionally, but structural use under development	x	0.667	0.67	0.67
4	Yes, we structurally use cyclic quality policy		1.000	0.00	1.00
				0.67	1.00

6.4 Does your diabetes outpatient clinic use a certified quality system?

	(choose the best possible answer)			experts' weighting	achieved weighted score	weighted maximum score
1	Yes, namely	x		1.000	1.00	1.00
2	ISO	x		0.000	0.00	0.00
3	INK			0.000	0.00	0.00
4	HKZ			0.000	0.00	0.00
5	NIAZ			0.000	0.00	0.00
6	Other namely		……………………	1.000	0.00	1.00
7	No			0.000	0.00	0.00
					1.00	1.00

6.5 Please indicate which of the following documents are available in your diabetes outpatient clinic?

		**No**	**Under development**	**Yes**	achieved weighted score	weighted maximum score
	(choose the best possible answer)	0.000	0.100	0.200		
1	**Mission document**: vision and priorities of the organisation			x	0.20	0.20
2	**Quality action plan** for organisation: measures for the implementation and planning of actions to achieve quality goals			x	0.20	0.20
3	**Annual quality report**: reporting on all activities carried out to ensure quality of care and its outcomes		x		0.10	0.20
4	**Quality manual**: description of all procedures used by the organisation and the individuals responsible to ensure quality of care			x	0.20	0.20
5	Is the quality manual available to all employees within the organisation?			x	0.20	0.20
					0.90	1.00

6.6 Which statement is most appropriate for the diabetes outpatient clinic?

	(choose the best possible answer)									
1	Our department uses quality documents of the entire hospital						0.500	0.00	0.50
2	Our department uses quality documents of the department of internal medicine	x					1.000	1.00	1.00
3	The diabetes outpatient clinic has its own quality documents						1.000	0.00	1.00
4	The diabetes clinic has no quality documents						0.000	0.00	0.00
								**1.00**	**1.00**

	Question number	score achieved	maximum points	achieved score		experts' weighting	achieved weighted score	weighted maximum score
Structural policy	6.1-6.3	2.17	3	72%		40%	29%	50%
Quality system	6.4	1.00	1	100%		40%	40%	17%
Quality documents	6.5 and 6.6	1.90	2	95%		20%	19%	33%
**TOTAL SCORE Management strategies**		**5.07**	**6**	84%		100%	88%	100%
